# Differences in predictors of incident heart failure according to atherosclerotic cardiovascular disease status

**DOI:** 10.1002/ehf2.14521

**Published:** 2023-09-09

**Authors:** Luke P. Dawson, Melinda J. Carrington, Tilahun Haregu, Shane Nanayakkara, Garry Jennings, Anthony Dart, Dion Stub, David Kaye

**Affiliations:** ^1^ Department of Cardiology The Alfred Hospital Melbourne Victoria Australia; ^2^ Faculty of Medicine Monash University Melbourne Victoria Australia; ^3^ Department of Cardiology The Royal Melbourne Hospital Melbourne Victoria Australia; ^4^ Baker Heart and Diabetes Institute 55 Commercial Rd, Prahran Melbourne Victoria Australia

**Keywords:** Risk factors, Heart failure, Cardiometabolic profile, Lifestyle, Socio‐economic status, Atherosclerotic cardiovascular disease

## Abstract

**Aims:**

Heart failure (HF) is a common cause of morbidity and mortality, related to a broad range of sociodemographic, lifestyle, cardiometabolic, and comorbidity risk factors, which may differ according to the presence of atherosclerotic cardiovascular disease (ASCVD). We assessed the association between incident HF with baseline status across these domains, overall and separated according to ASCVD status.

**Methods and results:**

We included 5758 participants from the Baker Biobank cohort without HF at baseline enrolled between January 2000 and December 2011. The primary endpoint was incident HF, defined as hospital admission or HF‐related death, determined through linkage with state‐wide administrative databases (median follow‐up 12.2 years). Regression models were fitted adjusted for sociodemographic variables, alcohol intake, smoking status, measures of adiposity, cardiometabolic profile measures, and individual comorbidities. During 65 987 person‐years (median age 59 years, 38% women), incident HF occurred among 784 participants (13.6%) overall. Rates of incident HF were higher among patients with ASCVD (624/1929, 32.4%) compared with those without ASCVD (160/3829, 4.2%). Incident HF was associated with age, socio‐economic status, alcohol intake, smoking status, body mass index (BMI), waist circumference, waist–hip ratio, systolic blood pressure (SBP), and low‐ and high‐density lipoprotein cholesterol (LDL‐C and HDL‐C), with non‐linear relationships observed for age, alcohol intake, BMI, waist circumference, waist–hip ratio, SBP, LDL‐C, and HDL‐C. Risk factors for incident HF were largely consistent regardless of ASCVD status, although diabetes status had a greater association with incident HF among patients without ASCVD.

**Conclusions:**

Incident HF is associated with a broad range of baseline sociodemographic, lifestyle, cardiometabolic, and comorbidity factors, which are mostly consistent regardless of ASCVD status. These data could be useful in efforts towards developing risk prediction models that can be used in patients with ASCVD.

## Introduction

Despite improvements in treatment strategies and mortality rates for heart failure (HF), prevalence remains high, and some studies suggest a rising incidence among younger adults in the United States.^1^, ^2^, ^3^, ^4^, ^5^ Potential contributing factors to ongoing high rates of HF hospitalization include an ageing population, rising rates of comorbidities, including cardiovascular risk factors such as hypertension, dyslipidaemia, and diabetes mellitus, and other population‐level changes in sociodemographic, lifestyle, or cardiometabolic profiles.^4^, ^5^ Improving prediction of incident HF may assist in reversing this trend by guiding population and individual preventative strategies, better understanding disease development, and facilitating early identification of HF among at‐risk patients before hospitalization. For example, early detection of HF through targeted screening among high‐risk groups may allow modification to the natural history of disease before patients decompensate and require admission to hospital.

Some studies have suggested that risk factors for incident HF may differ dependent on prior myocardial infarction status. However, existing prediction models for incident HF are largely targeted at individuals without any concomitant cardiovascular disease (CVD)^6^—excluding a group of patients who are at highest risk of HF. In this setting, it is timely to assess whether there are different risk profiles for incident HF among patients with and without established atherosclerotic cardiovascular disease (ASCVD), which in turn would support the development of separate prediction models for patients with ASCVD (in addition to existing models for the general population).

The present study sought to determine the relationship between a broad range of baseline sociodemographic, lifestyle, cardiometabolic, and comorbidity factors using longitudinal data from the Baker Biobank study overall and according to ASCVD status. Moreover, we aimed to appropriately account for the competing risk of non‐HF‐related death and assess for the presence of non‐linear relationships, which are increasingly recognized in the medical literature and are inconsistently accounted for in prior HF risk factor studies.^7^, ^8^, ^9^, ^10^, ^11^, ^12^, ^13^

## Methods

This study was approved by the Alfred Human Research Ethics Committee (EC 357/09), and approval for data linkage was provided by the Australian Institute of Health and Welfare Ethics Committee (EC/2009/4/51). All patients gave written, informed consent to participate in the study. The reporting of this study follows the Strengthening the Reporting of Observational Studies in Epidemiology (STROBE) reporting guidelines.

### Baker Biobank and study participants

The Baker Biobank is a CVD database in Australia that collected data on 6531 individuals aged 18–69 years between January 2000 and December 2011. Participants were recruited from hospital and health check clinics and included those with established CVD or with at least one risk factor for CVD (including obesity).^14^ The database includes baseline data regarding sociodemographic profile, medical history, family history, medication use, physical measurements, and biomarkers. Follow‐up data were determined via linkage with the Victorian Admitted Episodes Dataset (VAED), a state‐wide administrative database that includes discharge diagnostic data, and the National Death Index (NDI), including all admissions or deaths until May 2018. Further details regarding the Baker Biobank study methods are detailed in previous studies.^14^, ^15^, ^16^

Given that the purpose of the present study was to examine factors associated with development of incident HF, patients with a previous history of HF were excluded from the analysis. Patients not matched to the VAED or NDI datasets and therefore without any follow‐up data were also excluded.

### Baseline characteristics and atherosclerotic cardiovascular disease status

Baseline medical history, including comorbidities and medication history, was determined from patient self‐report and medical records at the time of the initial Baker Biobank consultation. Socio‐economic status was determined using the Index of Relative Socioeconomic Advantage and Disadvantage (IRSAD), derived from 2016 Australian Bureau of Statistics national census data. A percentile ranging from 0 (lowest) to 100 (highest) is calculated according to residential postcode based on household income, education level, unemployment rate, internet availability, and home and vehicle ownership. Alcohol intake (total standard drinks per week) and smoking status (never, previous, or current) were determined by participant self‐report. Baseline body mass index (BMI; kg/m^2^), waist measurements (cm), and waist–hip ratios were determined by registered nurses and/or trained study personnel according to standard procedures and definitions.^17^

Total cholesterol, low‐density lipoprotein cholesterol (LDL‐C), high‐density lipoprotein cholesterol (HDL‐C), triglycerides, and fasting glucose were measured immediately following baseline blood sampling as previously described.^14^ Systolic and diastolic blood pressures (SBP and DBP) were measured by study staff at the baseline study visit using the average of three recordings with an automated sphygmomanometer (Omron HEM‐907) in the seated position after 10 min of rest.

Patients were grouped according to ASCVD status defined as self‐reported myocardial infarction, coronary artery disease, previous coronary artery bypass graft surgery, previous coronary stenting, stroke, or peripheral vascular disease at baseline. Patients who had a linked admission for a diagnosis relating to ASCVD prior to an admission for HF were also included in the ASCVD group (see ASCVD admission definitions in Supporting Information, *Table* *S1*).

### Primary endpoint

The primary endpoint was incident HF defined as either (i) admission to hospital with HF recorded as a primary or non‐primary VAED diagnosis at discharge or (ii) death with HF recorded as a primary or non‐primary cause. HF hospitalization diagnosis in VAED and cause of death were defined according to International Classification of Diseases 10 Australian Modification (ICD‐10‐AM) with specific codes detailed in Supporting Information, *Table* *S2*.

### Statistical analyses

Missing data (Supporting Information, *Table* *S3*) were managed with multiple imputation with chained equations (20 imputations). All analyses were performed in each imputed dataset and results pooled using Rubin's rules. For the primary analyses, we used Fine–Gray sub‐distribution hazard models with non‐HF‐related death as a separate competing risk adjusted for age, sex, socio‐economic status, smoking status, alcohol intake, BMI, waist circumference, and individual comorbidities that were fitted for the primary endpoint (incident HF hospitalization or death related to HF). To facilitate visualization of results, continuous variables were categorized according to commonly used cut‐points. Models were fitted overall and separately according to ASCVD status. All hazard ratios (HRs) were plotted on log‐2 scales and no adjustments were made to account for multiple comparisons. We additionally adjusted for baseline use of any cholesterol‐lowering medications when assessing cholesterol profile, antihypertensive medications when assessing blood pressure measures, and diabetic medications when assessing glucose measures.

We performed several secondary analyses using Cox regression models. In these models, non‐HF‐related death was censored rather than included as a competing risk to facilitate the assessment of non‐linear effects and interactions according to ASCVD status. Models were adjusted for the same parameters as in the primary analysis. Continuous variables (age, socio‐economic status, alcohol quantity, BMI, waist circumference, waist–hip ratio, fasting glucose, SBP, DBP, total cholesterol, LDL‐C, HDL‐C, and triglycerides) were fitted as restricted cubic splines with three to five knots selected according to the number of knots minimizing the Akaike information criteria. The median value for the variable of interest was used as the reference point for plots of restricted cubic spline curves. Likelihood ratio tests were used to calculate *P*‐values for overall association (*P*‐overall) by comparing nested models with and without the variable of interest. *P*‐values for non‐linearity (*P*‐non‐linear) were calculated by comparing nested models with spline terms vs. nested models with linear terms. *P*‐values for interaction (*P*‐interaction) according to ASCVD status were calculated by comparing nested models with and without multiplicative interaction terms between ASCVD and the variable of interest.

Finally, we performed a sensitivity analysis repeating the primary analysis using a complete case approach to account for missing data rather than multiple imputation to assess the robustness of the primary results.

All analyses were conducted using R Version 4.2.2 using the *rms*, *ggplot2*, *ggsurvfit*, *tidycmprsk*, *hmisc*, *mice*, and *survival* packages.

## Results

In total, 6531 participants were included in the Baker Biobank. Participants without follow‐up data (71 people) and those with a history of HF (702 people) and those without follow‐up data were excluded, leaving 5758 participants in the analysis. Over a median follow‐up of 12.6 years [interquartile range (IQR) 8.1–15.1 years], representing 65 987 person‐years of follow‐up, incident HF occurred in 784 (13.6%) participants.

Of the cohort, 1929 participants (33.5%) had a history of ASCVD, while 3829 participants (66.5%) did not. Rates of incident HF during follow‐up were 32.4% (624 participants) in the ASCVD group and 4.2% (160 participants) in the non‐ASCVD group (*Table* *1*).

**Table 1:** Baker Biobank cohort characteristics

	ASCVD cohort		Non‐ASCVD cohort	
	Incident HF		Incident HF	
	Yes	No	Yes	No
Patients	624 (32.4)	1305 (67.7)	160 (4.2)	3669 (95.8)
Age	71 (64–78)	63 (54–71)	67 (58–75)	54 (43–64)
Sex				
Female	191 (30.6)	319 (24.4)	73 (45.6)	1615 (44.0)
Male	433 (69.4)	986 (75.6)	87 (54.4)	2054 (56.0)
Body mass index	27 (24–31)	27 (25–30)	27 (24–29)	26 (24–29)
Waist circumference	97 (88–106)	95 (88–104)	91 (86–101)	91 (81–100)
Waist–hip ratio	0.93 (0.89–0.98)	0.93 (0.89–0.97)	0.92 (0.87–0.96)	0.89 (0.82–0.94)
Smoking				
Never	239 (38.6)	541 (41.7)	82 (51.3)	2264 (61.9)
Previous	64 (10.3)	157 (12.1)	17 (10.6)	356 (9.7)
Current	316 (51.1)	601 (46.3)	61 (38.1)	1040 (28.4)
Alcohol, SD/week	0 (0–4)	1 (0–7)	1 (0–4)	2 (0–7)
Index of relative socio‐economic advantage and disadvantage	86 (54–94)	86 (55–94)	85 (54–95)	88 (65–95)
Comorbidities				
Hypertension	391 (62.7)	649 (50.0)	82 (51.9)	1102 (30.6)
Dyslipidaemia	352 (56.6)	769 (59.7)	50 (31.5)	1120 (31.8)
Diabetes mellitus	153 (25.8)	208 (17.5)	37 (28.0)	223 (8.0)
Coronary disease	523 (52.1)	735 (57.0)	N/A	N/A
Myocardial infarction	269 (43.4)	582 (44.8)	N/A	N/A
Valvular heart disease	76 (12.3)	55 (4.3)	19 (12.2)	80 (2.2)
Arrhythmia	142 (23.1)	236 (18.7)	39 (26.4)	508 (15.2)
Prior PCI	157 (25.2)	483 (37.2)	N/A	N/A
Prior CABG	182 (29.2)	303 (23.3)	N/A	N/A
Vascular disease	112 (18.1)	333 (25.8)	N/A	N/A
Stroke	84 (13.5)	126 (9.7)	N/A	N/A
Chronic kidney disease	60 (9.7)	65 (5.0)	7 (4.4)	88 (2.4)
Obstructive sleep apnoea	20 (3.2)	123 (3.5)	6 (3.8)	123 (3.5)
Asthma	92 (14.8)	172 (13.3)	27 (17.1)	515 (14.2)
Arthritis	89 (14.3)	193 (14.9)	21 (13.4)	470 (13.2)
Marfan's disease	2 (0.3)	2 (0.2)	0 (0)	5 (0.2)
Migraines	22 (3.5)	88 (6.8)	12 (7.6)	272 (7.5)
Retinopathy	56 (9.0)	93 (7.2)	9 (5.6)	147 (4.1)
Depression	33 (5.3)	92 (7.1)	10 (6.3)	299 (8.3)
Metabolic profile				
Systolic blood pressure	140 (125–150)	131 (120–145)	140 (130–150)	130 (120–142)
Diastolic blood pressure	80 (70–84)	80 (70–85)	80 (70–85)	80 (70–86)
Fasting glucose	5.6 (5.0–7.0)	5.5 (5.0–6.3)	5.3 (4.8–6.3)	5.2 (4.7–5.7)
Low‐density lipoprotein cholesterol	2.4 (1.9–3.1)	2.6 (2.0–3.2)	2.8 (2.2–3.4)	3.0 (2.4–3.62)
High‐density lipoprotein cholesterol	1.2 (0.9–1.5)	1.2 (0.9–1.4)	1.3 (1.1–1.6)	1.4 (1.1–1.7)
Triglycerides	1.5 (1.0–2.2)	1.5 (1.1–2.2)	1.4 (1.1–2.0)	1.3 (0.8–1.9)
Medications at baseline				
Cholesterol‐lowering agent	350 (56.1)	823 (63.1)	43 (26.9)	581 (15.8)
Antihypertensive	455 (72.9)	867 (66.4)	88 (55.0)	851 (23.2)
OHA or insulin	78 (12.5)	100 (7.7)	18 (11.3)	96 (2.6)

ASCVD, atherosclerotic cardiovascular disease; CABG, coronary artery bypass graft; HF, heart failure; N/A, not applicable; OHA, oral hypoglycaemic agent; PCI, percutaneous intervention; SD, standard drinks.

Data represent *n* (%) for categorical data and median (interquartile range) for continuous data.

Median age at enrolment was 59 years (IQR 47–69 years), and 38% were women. Participants who developed incident HF were older at enrolment (median 70 vs. 57 years) and more commonly male (66% vs. 61%). Differences in baseline characteristics between patients with ASCVD and those without ASCVD according to incident HF status are shown in *Table* *1*.

### Sociodemographic profile, lifestyle factors, and comorbidities

**Figure 1:**
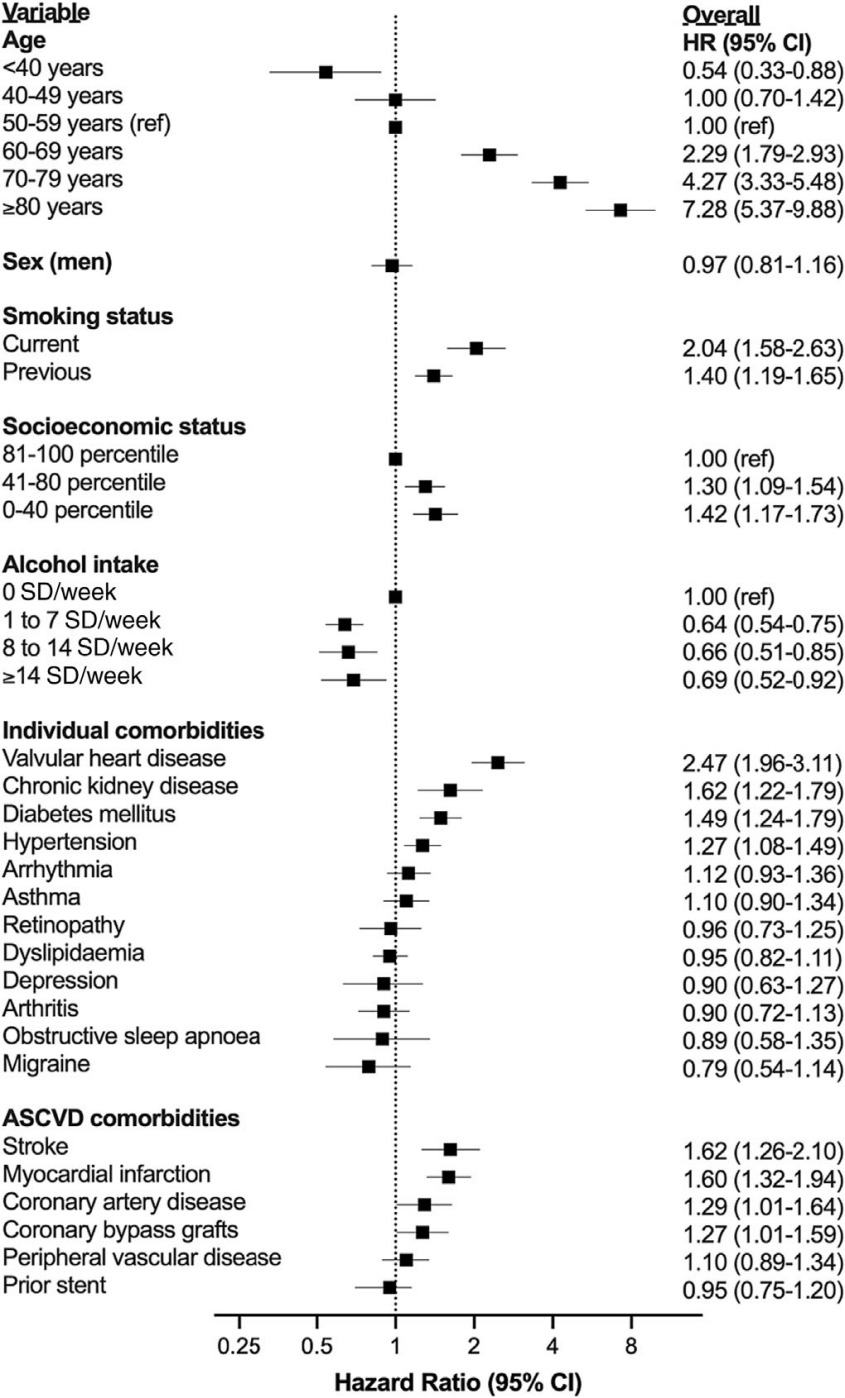
Hazard ratios (HRs) for incident heart failure (HF) over median 12.2 years of follow‐up. Sub‐distribution hazard models (Fine–Gray) accounting for non‐HF‐related death as a competing risk. ASCVD, atherosclerotic cardiovascular disease; CI, confidence interval; SD, standard drinks.

In adjusted Fine–Gray sub‐distribution hazard models, older age, lower socio‐economic status, current or previous smoking status, valvular heart disease, chronic kidney disease, diabetes mellitus, hypertension, stroke, myocardial infarction, coronary artery disease, and coronary bypass grafts were independently associated with risk of incident HF, while alcohol intake was inversely associated with incident HF risk (*Figure* *1*).

**Figure 2:**
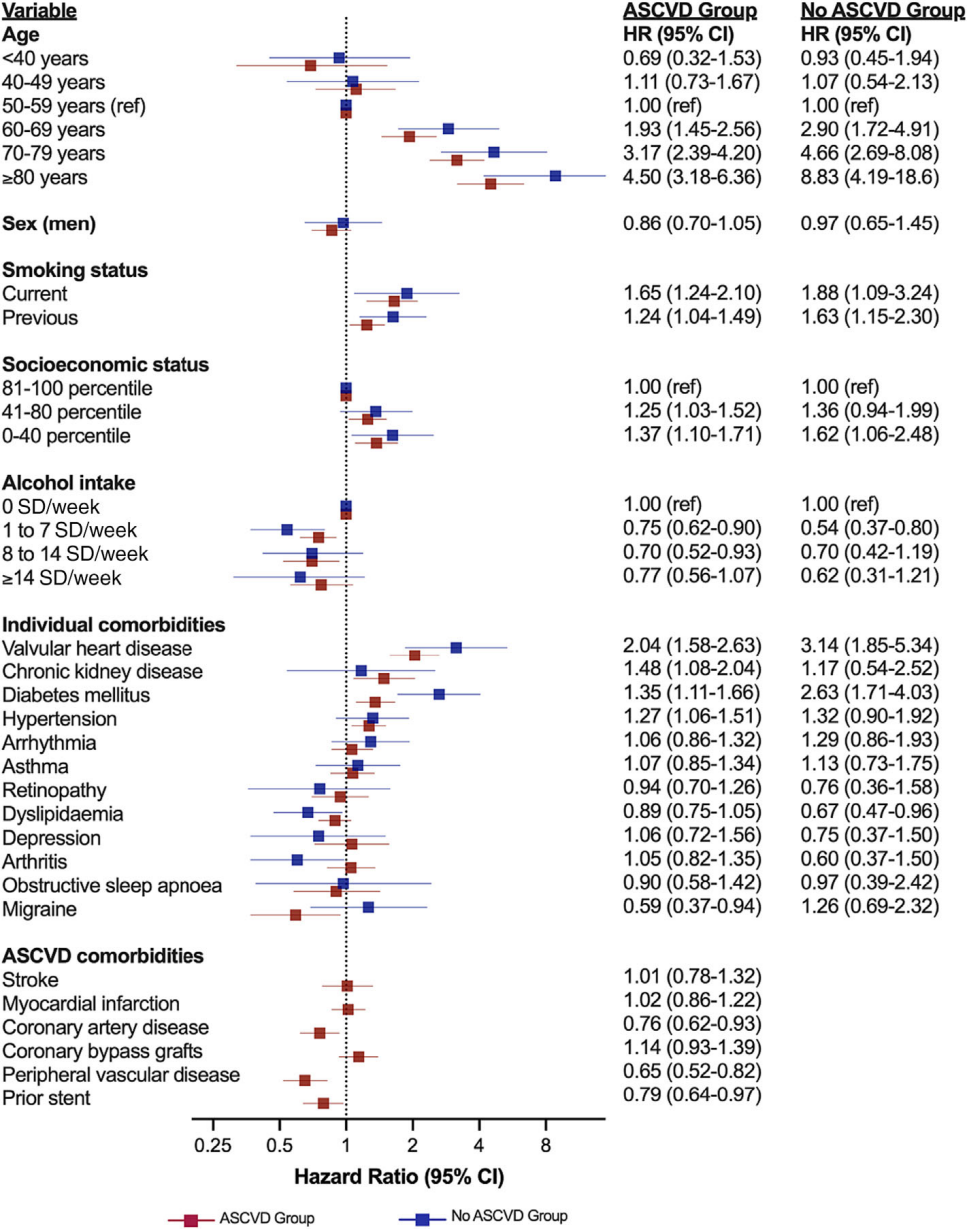
Hazard ratios (HRs) for incident heart failure (HF) according to atherosclerotic cardiovascular disease (ASCVD) status. Sub‐distribution hazard models (Fine–Gray) accounting for non‐HF‐related death as a competing risk. Red plots represent the HRs for patients with ASCVD, while blue plots indicate HRs for patients without ASCVD. CI, confidence interval; SD, standard drinks.

Minor differences in risk factors were present according to ASCVD status (*Figure* *2*). Specifically, diabetes mellitus was associated with a greater risk of incident HF among patients without ASCVD [adjusted HR (aHR) 2.63, 95% confidence interval (CI) 1.71–4.03] compared with those with ASCVD (aHR 1.35, 95% CI 1.11–1.66).

### Cardiometabolic profile

**Figure 3:**
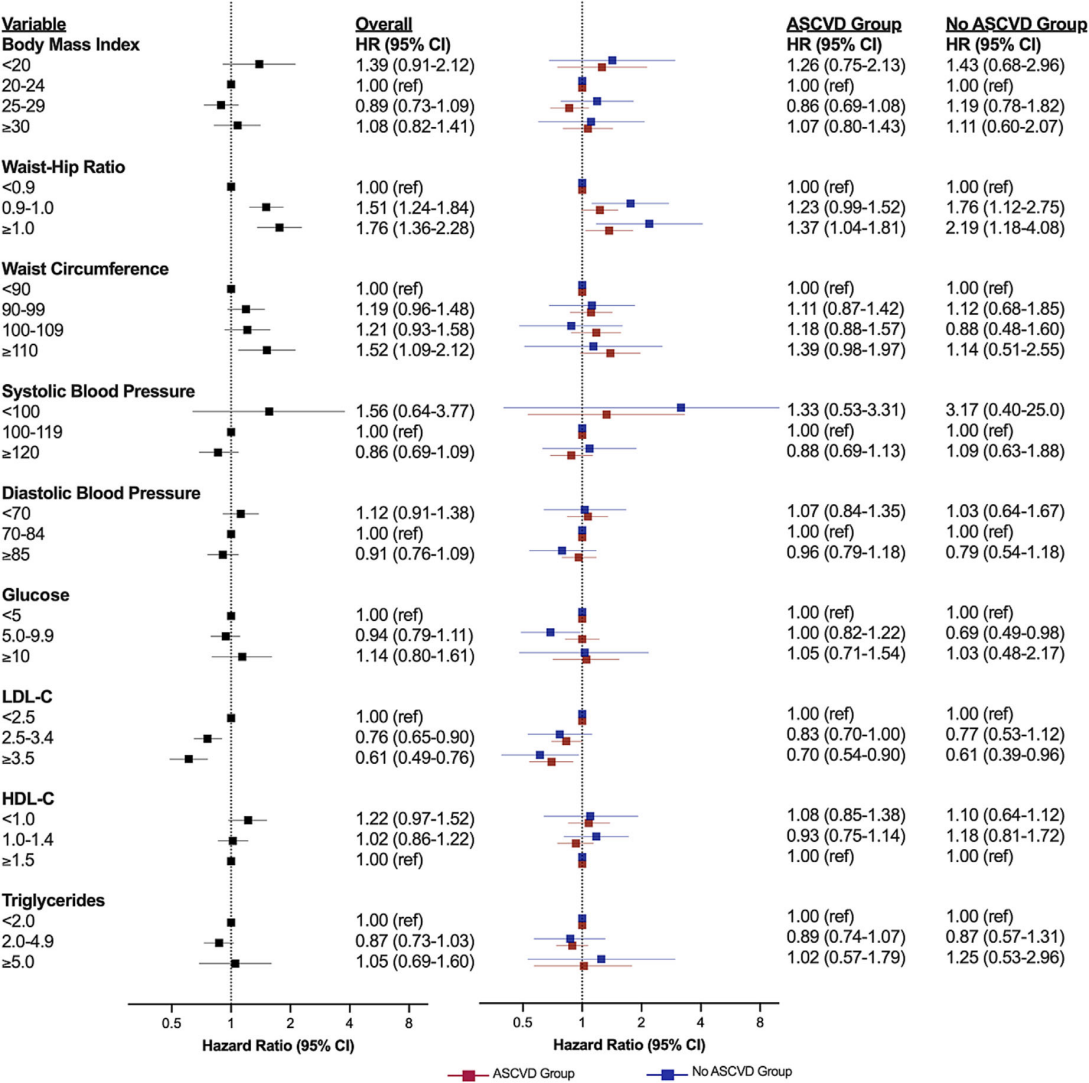
Cardiometabolic profile and risk of incident heart failure (HF) overall and according to atherosclerotic cardiovascular disease (ASCVD) status. Sub‐distribution hazard models (Fine–Gray) accounting for non‐HF‐related death as a competing risk. Red plots represent the hazard ratios (HRs) for patients with ASCVD, while blue plots indicate HRs for patients without ASCVD. CI, confidence interval; HDL‐C, high‐density lipoprotein cholesterol; LDL‐C, low‐density lipoprotein cholesterol.

In the primary analysis, greater waist–hip ratio and waist circumference were associated with higher risk of incident HF, and greater LDL‐C was inversely associated with incident HF risk (*Figure* *3*). Associations were not observed for SBP or DBP, glucose, and triglycerides. No significant differences were observed according to ASCVD status.

### Secondary analyses

**Figure 4:**
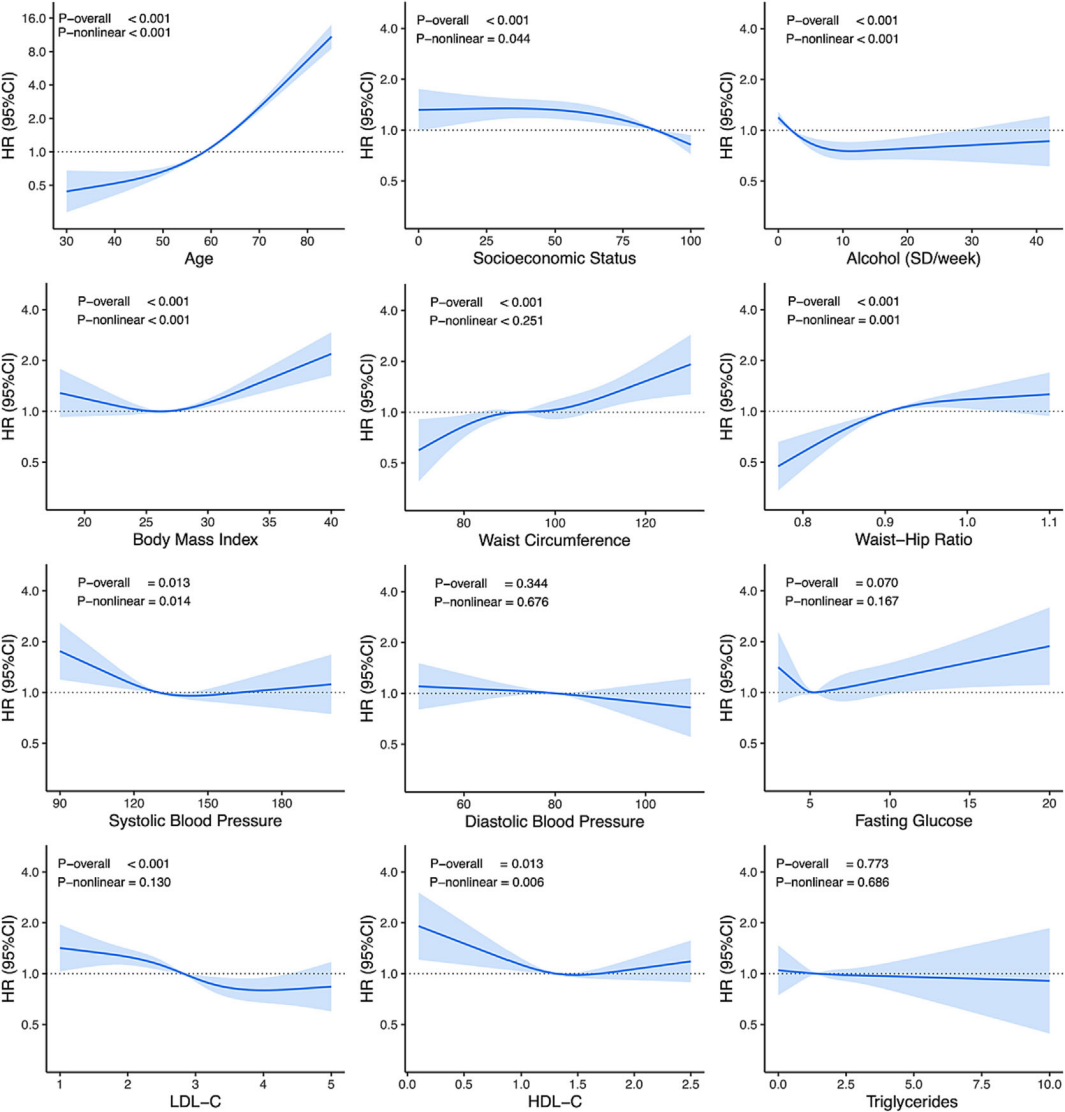
Secondary analyses assessing for non‐linearity in incident heart failure risk according to baseline sociodemographic, lifestyle, and cardiometabolic profiles. Cox models adjusted for age, sex, socio‐economic status, smoking status, alcohol intake, waist circumference*, body mass index*, systolic blood pressure*, low‐density lipoprotein cholesterol (LDL‐C)*, individual comorbidities, and baseline medication use with the variable of interest included as a restricted cubic spline. *P*‐overall indicates results of comparing nested models with and without the variable of interest using likelihood ratio tests, while *P*‐non‐linear indicates results of comparing nested models with spline terms vs. with linear terms for the variable of interest. *Variables excluded when main variable of interest is collinear [i.e. body mass index and waist circumference excluded for waist–hip ratio, systolic blood pressure excluded for diastolic blood pressure, and LDL‐C excluded for high‐density lipoprotein cholesterol (HDL‐C) and triglycerides]. CI, confidence interval; HR, hazard ratio; SD, standard drinks.

Secondary analyses assessing overall associations and non‐linearity for continuous sociodemographic and cardiometabolic variables using Cox regression models and restricted cubic splines are shown in *Figure* *4*. Of the continuous variables assessed, evidence of a non‐linear relationship was present for age, alcohol intake, and socio‐economic status (both *P*‐non‐linear < 0.05). Lower socio‐economic status was associated with increased risk below the median IRSAD percentile (87th percentile) and reduced risk above the median. Zero alcohol intake appeared to be associated with greater incident HF risk, while mild–moderate intake was associated with reduced risk.

BMI, waist circumference, waist–hip ratio, SBP, LDL‐C, and HDL‐C were each associated with incident HF risk in adjusted Cox models, while DBP, fasting glucose, and triglycerides were not. Evidence for non‐linear associations was present for BMI, waist–hip ratio, SBP, and HDL‐C (each *P*‐non‐linear < 0.05). For BMI, incident HF risk was greater above the median (27 kg/m^2^) but not reduced below the median, while for waist circumference, risk increased in a more proportional manner. SBP appeared to be inversely associated with HF risk, with higher risk among patients with lower baseline blood pressure. Risk of incident HF was higher for patients with lower baseline values for LDL‐C and HDL‐C.

**Figure 5:**
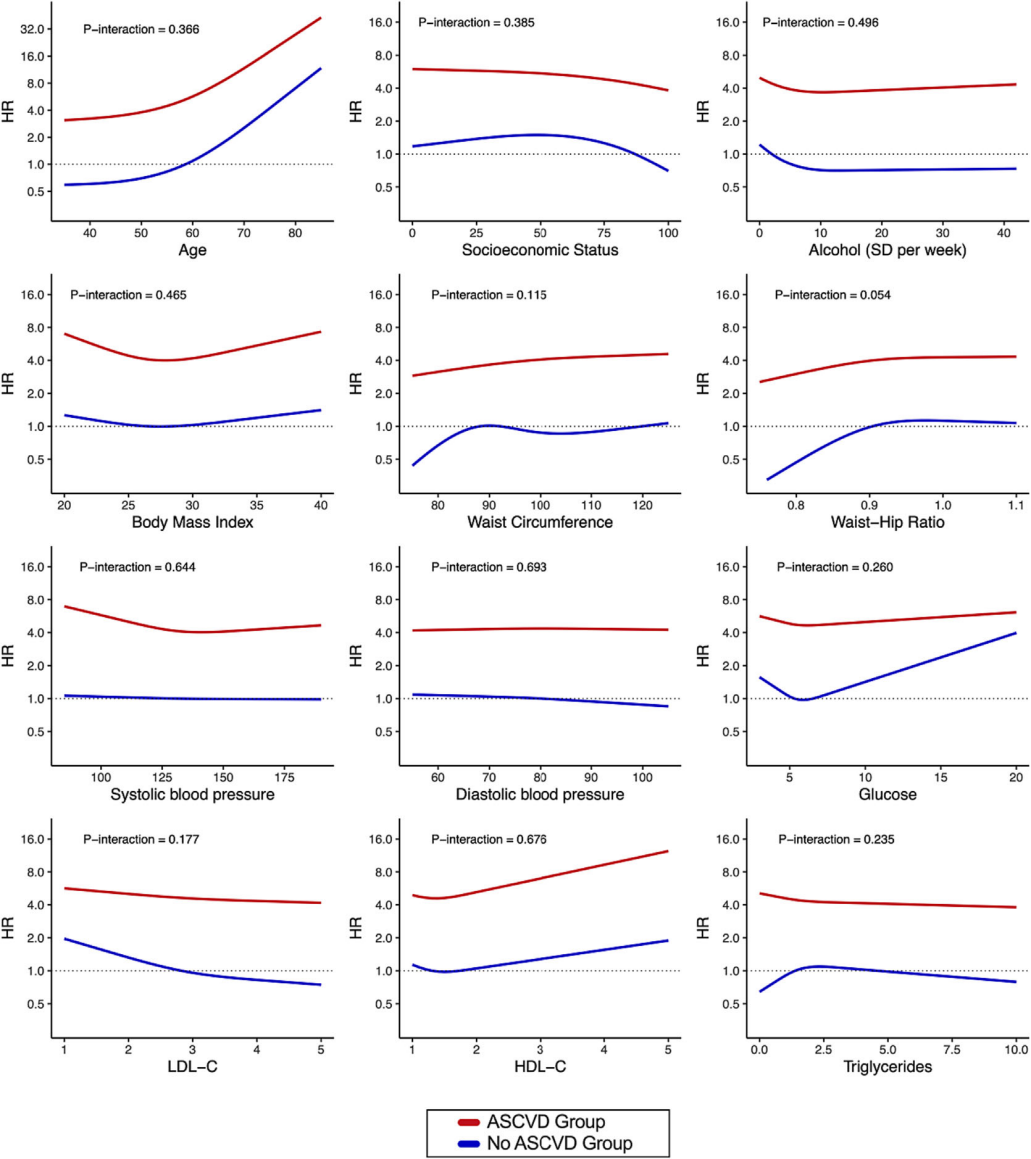
Secondary analyses assessing for interactions between baseline sociodemographic, lifestyle, and cardiometabolic profiles and atherosclerotic cardiovascular disease (ASCVD) status. Blue lines indicate hazard ratios (HRs) for incident heart failure among the non‐ASCVD group, while red lines indicate HRs among the ASCVD group. While incident heart failure risk was higher among the ASCVD cohort, no interactions were present according to baseline sociodemographic, lifestyle, and cardiometabolic profiles. Cox models adjusted for the same parameters as in *Figure* *4*. *P*‐interaction indicates results of comparing nested models with and without a multiplicative interaction term between the variable of interest using likelihood ratio tests, while *P*‐non‐linear indicates results of comparing nested models with spline terms and ASCVD status. HDL‐C, high‐density lipoprotein cholesterol; LDL‐C, low‐density lipoprotein cholesterol; SD, standard drinks.

Secondary analyses assessing interactions according to ASCVD status are shown in *Figure* *5*. Risk of incident HF was higher in the presence of ASCVD, but no significant interactions were observed across the various sociodemographic or cardiometabolic variables.

### Sensitivity analysis

Sensitivity analysis using a complete case approach to manage missing data rather than multiple imputation is shown in Supporting Information, *Figures* *S1*–*S3*. Results were comparable with the primary analysis.

## Discussion

In this prospective cohort of middle‐aged and older adults free from HF at baseline, comprising 65 987 person‐years at risk, incident HF was associated with a broad range of baseline sociodemographic, lifestyle, cardiometabolic, and comorbidity factors, including age, socio‐economic status, smoking status, alcohol intake, BMI, waist circumference, SBP, and cholesterol profile. Importantly, non‐linear associations were present for most of these variables. Risk factors for incident HF were largely consistent among patients with and without ASCVD, although effect modification was observed for diabetes mellitus, whereby a greater association with incident HF was present in the non‐ASCVD group for these variables. While some of these findings are confirmatory of previous results, this study provides a number of new insights through the breadth of baseline variables assessed. Moreover, these data could be useful in efforts to develop HF prediction models that can be used for patients with ASCVD, whether these are developed as part of models used in the general population (with additional variables) or as separate ASCVD‐specific models.

The influence of sociodemographic and lifestyle factors on HF development has been widely assessed.^18^, ^19^ HF incidence clearly becomes greater with increasing age, and our study suggests that there is an increase in the gradient of this risk at around 55–60 years, independent of other baseline factors. In previous studies, an inconsistent association between male sex and incident HF risk has been observed—a finding that was not present in our study, perhaps relating to greater adjustment for other baseline variables in comparison with previous studies.^20^ The association between socio‐economic inequity with risk of overall mortality, cardiovascular mortality, and CVD incidence is well established,^21^ and our results confirm that this relationship extends to HF incidence. Most prior analyses have relied on using categorized measures of socio‐economic status rather than a continuous measure such as percentile, and our data suggest that aHRs remained relatively constant below the 70th percentile. Moreover, this relationship was present after adjustment for individual‐level lifestyle, cardiometabolic, and comorbidity factors, suggesting that other factors relating to socio‐economic status play a role in HF incidence, for example, access to care, primary prevention measures, and other systemic social determinants of health. Smoking status was strongly associated with incident HF risk, and the difference in HRs between current (HR 2.04) and previous (HR 1.40) smokers suggests an important role for encouraging smoking cessation as a preventative measure for subsequent HF. Most studies assessing the influence of alcohol intake on incident HF have identified a lower risk of HF among modest drinkers compared with non‐drinkers, with suggested mechanisms including beneficial effects on lipid profiles, insulin sensitivity, or inflammatory state, or residual confounding.^7^, ^9^, ^21^, ^22^ Prior studies have mostly relied on categorizing alcohol intake and our study further defines this relationship by assessing non‐linear effects with alcohol as a continuous variable.

Most measures of cardiometabolic profile were associated with incident HF risk. Multiple studies have identified a relationship between measures of obesity and body fat and incident HF risk, but the non‐linearity of this relationship is not well established.^11^, ^17^, ^23^, ^24^ Our study suggests that there is a J‐curve relationship between BMI and incident HF. Proposed mechanisms between adiposity and incident HF risk include associations with the metabolic syndrome (although these were adjusted for in our analyses), a proinflammatory state, impaired cardiorespiratory fitness, increased haemodynamic load, and adverse effects on systolic and diastolic function.^11^, ^17^, ^24^, ^25^ Mechanisms underlying the increased risk with low BMI are unclear, which might be representative of an early disease state leading to weight loss rather than low BMI leading to disease. Lower but not higher baseline SBP measures were associated with increased HF risk, which appeared to be limited to patients on blood pressure‐lowering medications in interactive analysis, and might reflect an early disease state leading to lower blood pressure rather than a causative mechanism (especially given that hypertension and other individual comorbidities comprising the metabolic syndrome were associated with incident HF).

The limited differences in risk factor significance between patients with ASCVD and those without ASCVD were somewhat surprising and contrast previous data regarding patients with and without a history of myocardial infarction. Among people hospitalized with HF, 56% have a comorbid diagnosis of ischaemic heart disease,^26^ highlighting the importance of developing risk prediction tools that can be used for this group. Prior risk prediction models have largely been focused on incident HF risk in the general population. Given that there were only minor differences in risk factors between the ASCVD and non‐ASCVD cohorts, risk models aimed at predicting incident HF for patients with ASCVD could be developed by including additional variables to models aimed at the general population (e.g. presence or absence of ASCVD) or by developing separate models for use in ASCVD cohorts alone. If the former approach is used, our data suggest that it is worthwhile considering a potential interactive effect between ASCVD and diabetic status.

### Limitations

This study has several limitations. The Baker Biobank is not fully representative of the wider Australian population. For example, a greater number of participants had high socio‐economic status according to postcode‐derived IRSAD percentile compared with the general population. Some lifestyle variables, such as exercise quantity and dietary factors, were not available in the dataset, hence limiting the assessment of lifestyle measures to alcohol quantity and smoking status. The study also relied on associations for individual measures at baseline only and data were not collected on changes in these factors across serial visits.^27^ HF hospitalizations defined using ICD codes could not be categorized according to HF with preserved vs. reduced ejection fraction (HFpEF vs. HFrEF), which have different risk factor profiles.^28^ Incorporation of ejection fraction data into hospitalization datasets (or amendment of future ICD coding versions to delineate HFpEF and HFrEF phenotypes) in future studies would be highly valuable. Similarly, HF hospitalization is representative of acute care for HF but does not capture incident HF managed as an outpatient. If patients included in the study were managed differently in the ambulatory setting based on their risk factor profiles, this may have led to biases in HF admission rates, which would influence the results. Finally, while we aimed to account for non‐HF‐related death as a competing risk in the primary analyses using Fine–Gray hazard models to avoid overestimation of HRs,^29^ we were unable to easily plot non‐linear relationships using this approach and, therefore, this analysis was limited to categorization of the variable of interest.

## Conclusions

In this prospective cohort study of middle‐aged and older adults free from HF at baseline, incident HF was associated with a broad range of baseline sociodemographic, lifestyle, cardiometabolic, and comorbidity factors, including age, socio‐economic status, smoking status, alcohol intake, BMI, waist circumference, SBP, and cholesterol profile. Risk factors were largely comparable between patients with ASCVD and those without ASCVD, although diabetes status had a greater association with incident HF among patients without ASCVD. These findings highlight the broad range of factors that should be considered in developing improved population‐ and individual‐level preventative strategies for incident HF and could be useful in efforts towards developing risk prediction models that can be used in patients with ASCVD.

## Conflict of interest

L.D. is supported by National Health and Medical Research Council of Australia (NHMRC) and National Heart Foundation (NHF) postgraduate scholarships. D.K. and D.S. are supported by NHF and NHMRC grants. M.J.C. receives an endowed fellowship in the Cardiology Centre of Excellence from Filippo and Maria Casella.

## Funding

The study was supported by a programme grant from the National Health and Medical Research Council.

## Supporting information


**Table S1.** International Classification of Disease 10 definitions for admissions relating to atherosclerotic cardiovascular disease.
**Table S2.** International Classification of Disease 10 definitions for heart failure incidence.
**Table S3.** Rates of missing baseline data in the Baker Biobank dataset.
**Figure S1.** Sensitivity analysis using complete case approach for Figure 1.
**Figure S2.** Sensitivity analysis using complete case approach for Figure 2.
**Figure S3.** Sensitivity analysis using complete case approach for Figure 3.
**Methods S1.** R code used for analyses.
