# Supplementary material for: Differences in predictors of incident heart failure according to atherosclerotic cardiovascular disease status
Source: ESC Heart Fail. 2023 Sep 9;10(6):3398–409. doi: 10.1002/ehf2.14521 (PMC10682860; doi:10.1002/ehf2.14521)
Supplement: Supplementary file 1 — Table S1. International Classification of Disease 10 definitions for admissions relating to atherosclerotic cardiovascular disease. Table S2. International Classification of Disease 10 definitions for heart failure incidence. Table S3. Rates of missing baseline data in the Baker Biobank dataset. Figure S1. Sensitivity analysis using complete case approach for Figure 1. Figure S2. Sensitivity analysis using complete case approach for Figure 2. Figure S3. Sensitivity analysis using complete case approach for Figure 3. Methods S1. R code used for analyses. [file EHF2-10-3398-s001.docx]

**Supplemental Material**

**Differences in predictors of incident heart failure according to atherosclerotic cardiovascular disease status**

Luke P. Dawson MBBS, MPH^a,b,c^, Melinda J Carrington, PhD,^d^ Tilahun Haregu, PhD,^a,d^ Shane Nanayakkara, MBBS, PhD,^a,d^ Garry Jennings, MD,^d^ Anthony Dart, BM, BCh, DPhil,^a,d^ Dion Stub,^a,b,d^ MBBS, PhD, David Kaye MBBS, PhD^a,b,d^

**eTable 1.** International Classification of Disease 10 definitions for heart failure incidence.

**eTable 2.** Rates of missing baseline data

**eFigure 1.** Sensitivity analysis using complete case approach for Figure 1.

**eFigure 2.** Sensitivity analysis using complete case approach for Figure 2.

**eFigure 3.** Sensitivity analysis using complete case approach for Figure 3.

**eMethods 1.** R code used for analyses

| **ICD-10** | **Description** |
| --- | --- |
| I210 | Acute transmural myocardial infarction of anterior wall |
| I211 | Acute transmural myocardial infarction of inferior wall |
| I212 | Acute transmural myocardial infarction of other sites |
| I213 | Acute transmural myocardial infarction of unspecified site |
| I214 | Acute subendocardial myocardial infarction |
| I219 | Acute myocardial infarction, unspecified |
| I220 | Subsequent myocardial infarction of anterior wall |
| I221 | Subsequent myocardial infarction of inferior wall |
| I228 | Subsequent myocardial infarction of other sites |
| I229 | Subsequent myocardial infarction of unspecified site |
| I230 | Haemopericardium as current complication following acute myocardial infarction |
| I231 | Atrial septal defect as current complication following acute myocardial infarction |
| I232 | Ventricular septal defect as current complication following acute myocardial infarction |
| I233 | Rupture of cardiac wall without haemopericardium as current complication following acute myocardial infarction |
| I234 | Rupture of chordae tendineae as current complication following acute myocardial infarction |
| I235 | Rupture of papillary muscle as current complication following acute myocardial infarction |
| I236 | Thrombosis of atrium, auricular appendage, and ventricle as current complications following acute myocardial infarction |
| I238 | Other current complications following acute myocardial infarction |
| I240 | Coronary thrombosis not resulting in myocardial infarction |
| I241 | Dresslers syndrome |
| I248 | Other forms of acute ischaemic heart disease |
| I249 | Acute ischaemic heart disease, unspecified |
| I250 | Atherosclerotic cardiovascular disease, so described |
| I2510 | Atherosclerotic heart disease, of unspecified vessel |
| I2511 | Atherosclerotic heart disease, of native coronary artery |
| I2512 | Atherosclerotic heart disease, of autologous bypass graft |
| I2513 | Atherosclerotic heart disease, of nonautologous bypass graft |
| I252 | Old myocardial infarction |
| I254 | Coronary artery aneurysm and dissection |
| I255 | Ischaemic cardiomyopathy |
| I256 | Silent myocardial ischaemia |
| I258 | Other forms of chronic ischaemic heart disease |
| I259 | Chronic ischaemic heart disease, unspecified |
| I630 | Cerebral infarction due to thrombosis of precerebral arteries |
| I631 | Cerebral infarction due to embolism of precerebral arteries |
| I632 | Cerebral infarction due to unspecified occlusion or stenosis of precerebral arteries |
| I633 | Cerebral infarction due to thrombosis of cerebral arteries |
| I634 | Cerebral infarction due to embolism of cerebral arteries |
| I635 | Cerebral infarction due to unspecified occlusion or stenosis of cerebral arteries |
| I636 | Cerebral infarction due to cerebral venous thrombosis, nonpyogenic |
| I638 | Other cerebral infarction |
| I639 | Cerebral infarction, unspecified |
| I650 | Occlusion and stenosis of vertebral artery |
| I651 | Occlusion and stenosis of basilar artery |
| I652 | Occlusion and stenosis of carotid artery |
| I653 | Occlusion and stenosis of multiple and bilateral precerebral arteries |
| I658 | Occlusion and stenosis of other precerebral artery |
| I659 | Occlusion and stenosis of unspecified precerebral artery |
| I660 | Occlusion and stenosis of middle cerebral artery |
| I661 | Occlusion and stenosis of anterior cerebral artery |
| I662 | Occlusion and stenosis of posterior cerebral artery |
| I663 | Occlusion and stenosis of cerebellar arteries |
| I664 | Occlusion and stenosis of multiple and bilateral cerebral arteries |
| I668 | Occlusion and stenosis of other cerebral artery |
| I669 | Occlusion and stenosis of unspecified cerebral artery |
| I672 | Cerebral atherosclerosis |
| I693 | Sequelae of cerebral infarction |
| I700 | Atherosclerosis of aorta |
| I701 | Atherosclerosis of renal artery |
| I7020 | Atherosclerosis of arteries of extremities, unspecified |
| I7021 | Atherosclerosis of arteries of extremities with intermittent claudication |
| I7022 | Atherosclerosis of arteries of extremities with rest pain |
| I7023 | Atherosclerosis of arteries of extremities with ulceration |
| I7024 | Atherosclerosis of arteries of extremities with gangrene |
| I708 | Atherosclerosis of other arteries |
| I709 | Generalised and unspecified atherosclerosis |
| I739 | Peripheral vascular disease, unspecified |

**eTable 1. International Classification of Disease 10 definitions for admissions relating to atherosclerotic cardiovascular disease.** ASCVD defined as present before HF if the following ICD-10 codes were recorded as primary of non-primary diagnosis in the Victorian Admitted Episodes Dataset (hospitalisation) or cause of death in the Victorian Death Index (death) prior to an admission or death related to HF (see eTable 2).

| **Heart failure incidence** | **ICD-10** | **Description** |
| --- | --- | --- |
| Incident heart failure defined as present if the following ICD-10 codes were recorded as primary of non-primary diagnosis in the Victorian Admitted Episodes Dataset (hospitalisation) or cause of death in the Victorian Death Index (death) | I110 | Hypertensive heart disease with (congestive) heart failure) |
|  | I130 | Hypertensive heart disease and kidney disease with (congestive) heart failure |
|  | I132 | Hypertensive heart disease and kidney disease with both (congestive) heart failure and kidney failure |
|  | I255 | Ischaemic cardiomyopathy |
|  | I420 | Dilated cardiomyopathy |
|  | I421 | Obstructive hypertrophic cardiomyopathy |
|  | I422 | Other hypertrophic cardiomyopathy |
|  | I426 | Alcoholic cardiomyopathy |
|  | I429 | Cardiomyopathy, unspecified |
|  | I431 | Cardiomyopathy in metabolic diseases |
|  | I500 | Congestive heart failure |
|  | I501 | Left ventricular failure |
|  | I509 | Heart failure, unspecified |
|  | I517 | Cardiomegaly |

**eTable 2. International Classification of Disease 10 definitions for heart failure incidence.**

|  | **Missing rates** |
| --- | --- |
| **Variable** | **n/N (%)** |
| Age | 7/5758 (0.1%) |
| Sex | 0/5758 (0%) |
| Socioeconomic status | 52/5758 (0.9%) |
| Alcohol intake | 205/5758 (3.6%) |
| Smoking status | 20/5758 (0.4%) |
| Body mass index | 358/5758 (6.2%) |
| Waist circumference | 946/5758 (16.4%) |
| Waist-hip ratio | 1161/5758 (20.3%) |
| Systolic blood pressure | 687/5758 (11.9%) |
| Diastolic blood pressure | 711/5758 (12.4%) |
| Glucose level | 351/5758 (6.1%) |
| LDL-C | 667/5758 (11.6%) |
| HDL-C | 330/5758 (5.7%) |
| Triglycerides | 309/5758 (5.4%) |
| Cholesterol lowering medications | 0/5758 (0%) |
| Antihypertensive medications | 0/5758 (0%) |
| Diabetic medications | 0/5758 (0%) |
| Hypertension | 75/5758 (1.3%) |
| Dyslipidaemia | 164/5758 (2.9%) |
| Stroke | 27/5758 (0.5%) |
| Myocardial infarction | 25/5758 (0.4%) |
| Depression | 84/5758 (1.5%) |
| Prior CABG | 9/5758 (0.2%) |
| Prior PCI | 17/5758 (0.3%) |
| Arrhythmia | 397/5758 (6.9%) |
| Coronary disease | 74/5758 (1.3%) |
| Vascular disease | 71/5758 (1.2%) |
| Valvular disease | 59/5758 (1.0%) |
| Kidney disease | 37/5758 (0.6%) |
| Obstructive sleep apnoea | 122/5758 (2.1%) |
| Asthma | 50/5758 (0.9%) |
| Arthritis | 116/5758 (2.0%) |
| Retinopathy | 68/5758 (1.2%) |
| Migraine | 52/5758 (0.9%) |
| Diabetes mellitus | 1060/5758 (18.4%) |
|  |  |

**eTable 3. Rates of missing baseline data in the Baker Biobank dataset.**

**
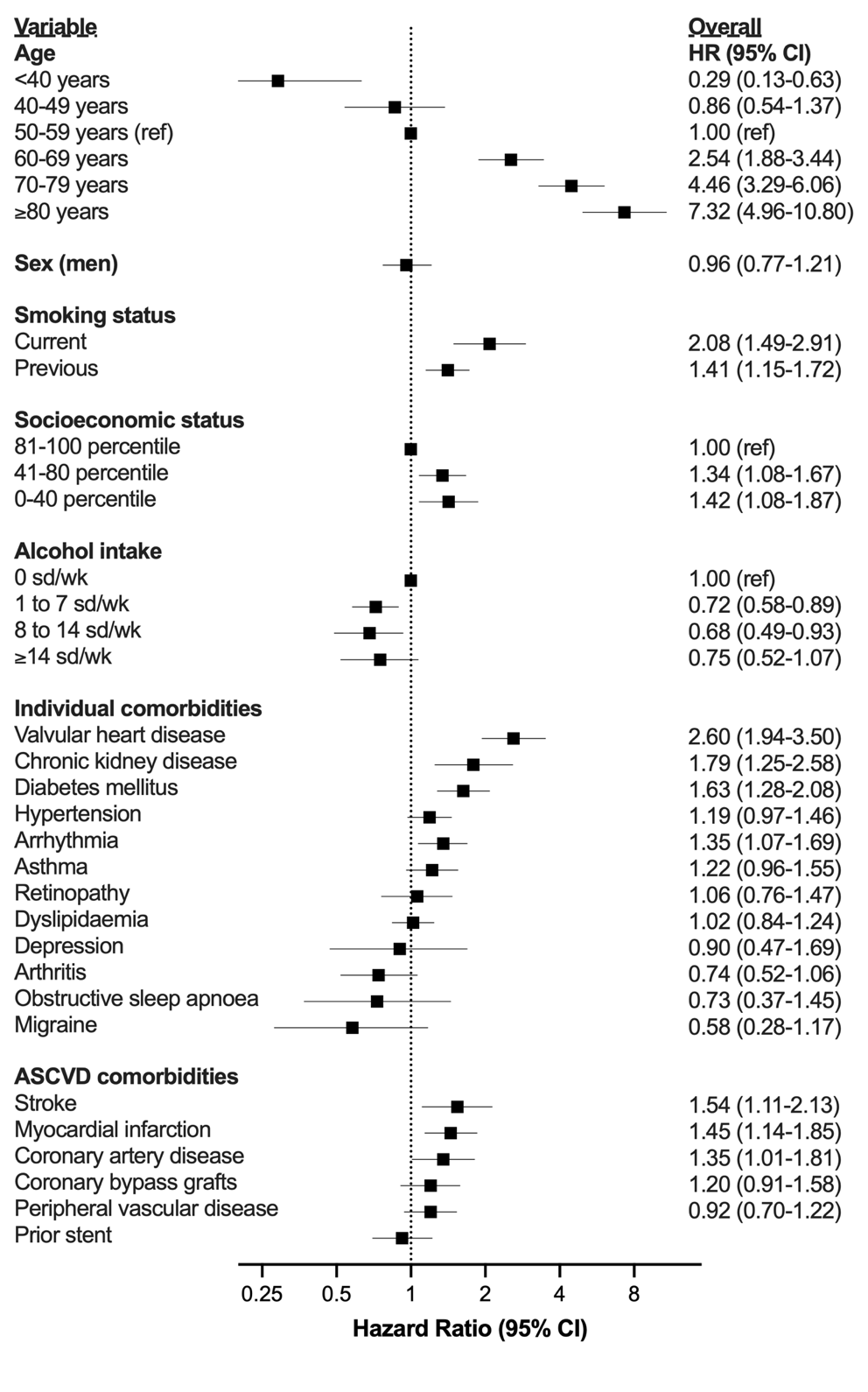
**

**eFigure 1.** Sensitivity analysis using complete case approach for Figure 1 (2060 cases omitted due to missing data).

**
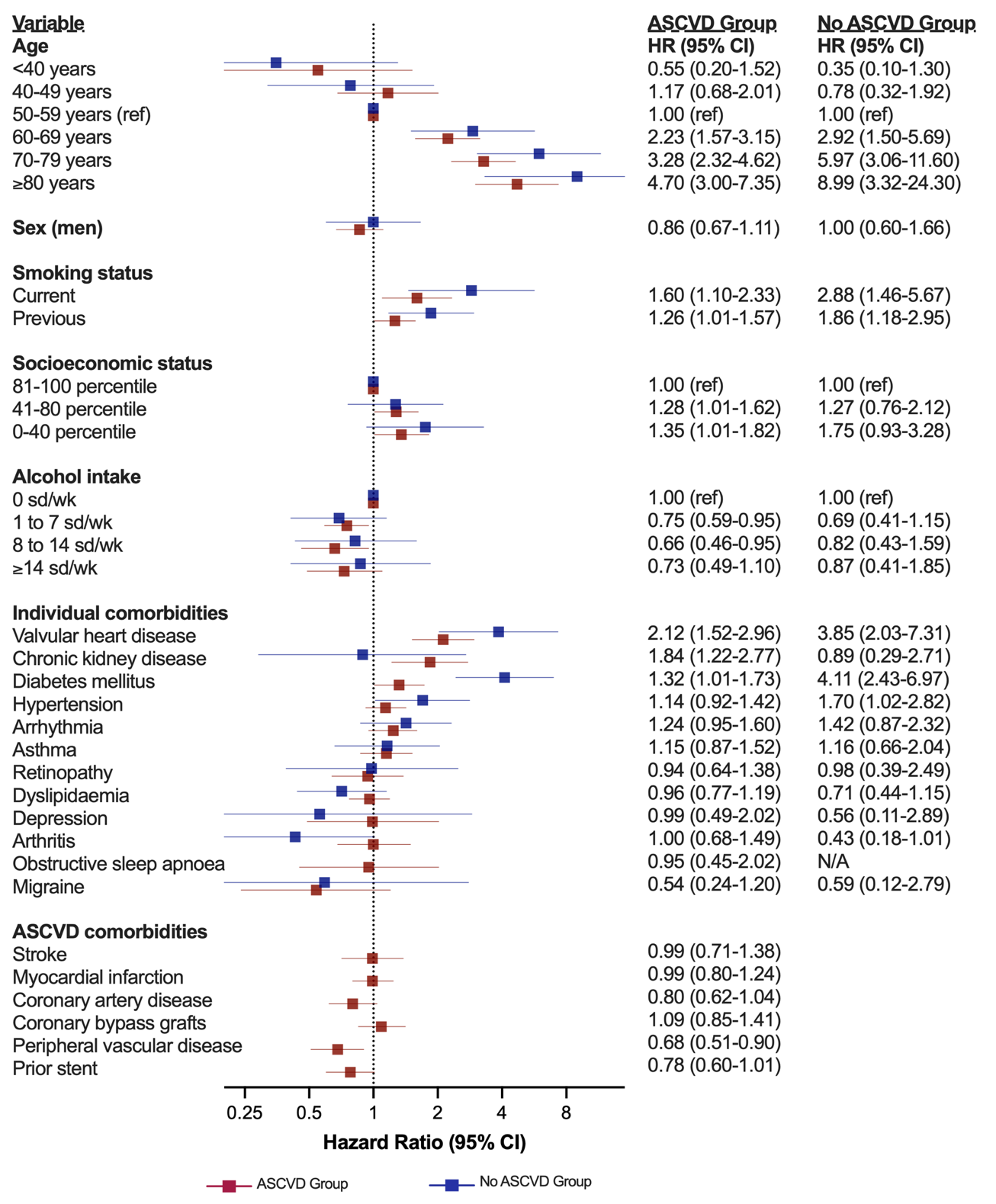
**

**eFigure 2.** Sensitivity analysis using complete case approach for Figure 2 (638 cases omitted from ASCVD group and 1421 cases omitted from non-ASCVD group due to missing data).

**
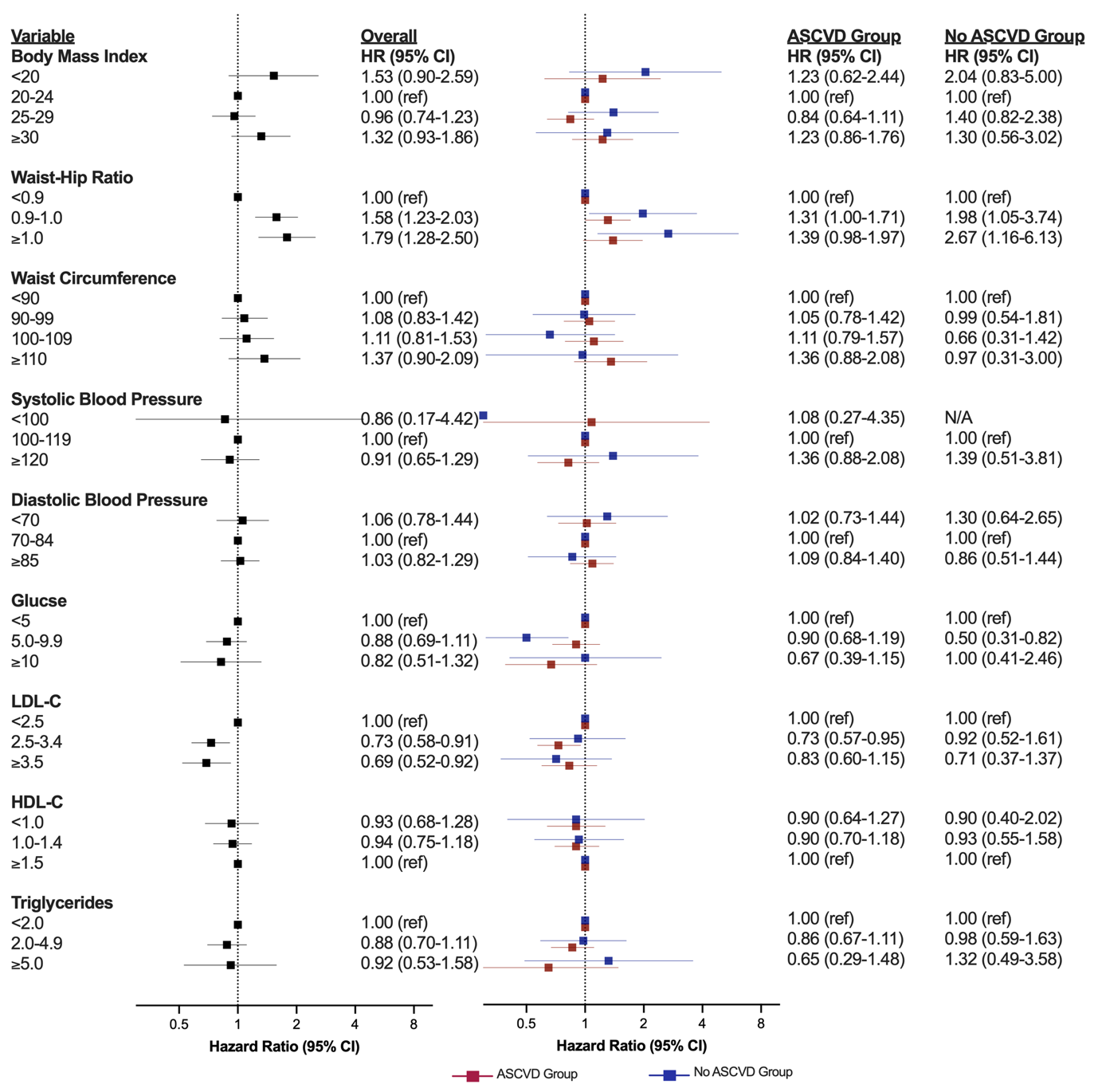
**

**eFigure 3.** Sensitivity analysis using complete case approach for Figure 3.

**eMethods 1. R Code used for analyses**

r-script-hf.R

lukedawson

2023-07-02

library(mice);library(rms);library(Hmisc);library(survival);library(ggplot2); library(tidycmprsk); library(dplyr)
options(scipen=999)

dd<-datadist(bkdata, adjto.cat='first')
options(datadist="dd")

bkdata$smoker<-as.factor(bkdata$smoker)
bkdata$sex<-as.factor(bkdata$sex)
bkdata$cholmed<-as.factor(bkdata$cholmed)
bkdata$dmmed<-as.factor(bkdata$dmmed)
bkdata$bpmed<-as.factor(bkdata$bpmed)
bkdata$hx_mi<-as.factor(bkdata$hx_mi)
bkdata$hx_stroke<-as.factor(bkdata$hx_stroke)
bkdata$hx_ht<-as.factor(bkdata$hx_ht)
bkdata$hx_chol<-as.factor(bkdata$hx_chol)
bkdata$hx_depression<-as.factor(bkdata$hx_depression)
bkdata$hx_cabg<-as.factor(bkdata$hx_cabg)
bkdata$hx_stent<-as.factor(bkdata$hx_stent)
bkdata$hx_arrhyth<-as.factor(bkdata$hx_arrhyth)
bkdata$hx_cad<-as.factor(bkdata$hx_cad)
bkdata$hx_vd<-as.factor(bkdata$hx_vd)
bkdata$hx_valve_dis<-as.factor(bkdata$hx_valve_dis)
bkdata$hx_kidney_dis<-as.factor(bkdata$hx_kidney_dis)
bkdata$hx_osa<-as.factor(bkdata$hx_osa)
bkdata$hx_asthma<-as.factor(bkdata$hx_asthma)
bkdata$hx_arthritis<-as.factor(bkdata$hx_arthritis)
bkdata$hx_retinopathy<-as.factor(bkdata$hx_retinopathy)
bkdata$hx_migraine<-as.factor(bkdata$hx_migraine)
bkdata$hx_marfan<-as.factor(bkdata$hx_marfan)
bkdata$hx_diab_any_any<-as.factor(bkdata$hx_diab_any_any)
bkdata$mort<-as.factor(bkdata$mort)
bkdata$hfmort<-as.factor(bkdata$hfmort)
bkdata$hfany <-as.factor(bkdata$hfany)
bkdata$ascvd <-as.factor(bkdata$ascvd)
bkdata$irsadpercent<-as.numeric(bkdata$irsadpercent)
bkdata$alcoholqty<-as.numeric(bkdata$alcoholqty)
bkdata$dbp_mean<-as.numeric(bkdata$dbp_mean)
bkdata$sbp_mean<-as.numeric(bkdata$sbp_mean)
bkdata$hfcomposite <-as.factor(bkdata$hfcomposite)

impbkdata<-mice(bkdata,m=20,maxit=10,seed=1234)

complete.impbkdata<-complete(impbkdata,action="long",include=TRUE)

#CHANGE OUTCOME BACK TO NUMERIC

bkdata$mort<-as.numeric(bkdata$mort)
bkdata$hfmort<-as.numeric(bkdata$hfmort)
bkdata$hfany <-as.numeric(bkdata$hfany)
bkdata$hfcomposite <-as.factor(bkdata$hfcomposite)
bkdata$morbcount <-as.factor(bkdata$morbcount)

complete.impbkdata$mort<-as.numeric(complete.impbkdata$mort)
complete.impbkdata$hfmort<-as.numeric(complete.impbkdata$hfmort)
complete.impbkdata$hfany <-as.numeric(complete.impbkdata$hfany)
complete.impbkdata$hfcomposite <-as.factor(complete.impbkdata$hfcomposite)
complete.impbkdata$morbcount <-as.factor(complete.impbkdata$morbcount)
impbkdata<-as.mids(complete.impbkdata)

#PRIMARY ANALYSIS SUBDISTRIBUTION MODELS IN MULTIPLY IMPUTED DATASET

complete.impbkdata<-complete(impbkdata,action="long",include=TRUE)
complete.impbkdata$agecat<-cut(complete.impbkdata$age, breaks=c(0,40,50,60,70,80,150),right=FALSE)
complete.impbkdata$agecat<-relevel(complete.impbkdata$agecat,ref=3)
complete.impbkdata$irsadpercentcat<-cut(complete.impbkdata$irsadpercent, breaks=c(-1,40,80,101))
complete.impbkdata$irsadpercentcat<-relevel(complete.impbkdata$irsadpercentcat,ref=3)
complete.impbkdata$alcoholqtycat<-cut(complete.impbkdata$alcoholqty, breaks=c(-1,0,7,14,1500))
complete.impbkdata$bmicat<-cut(complete.impbkdata$BMI_trim, breaks=c(0,20,25,30,100),right=FALSE)
complete.impbkdata$bmicat<-relevel(complete.impbkdata$bmicat,ref=2)
complete.impbkdata$waistcat<-cut(complete.impbkdata$waist_trim, breaks=c(0,90,100,110,1000),right=FALSE)
complete.impbkdata$waistcat<-relevel(complete.impbkdata$waistcat,ref=1)
complete.impbkdata$whrcat<-cut(complete.impbkdata$WHR_trim, breaks=c(0,0.90,1.00,10),right=FALSE)
complete.impbkdata$whrcat<-relevel(complete.impbkdata$whrcat,ref=1)
complete.impbkdata$sbpcat<-cut(complete.impbkdata$sbp_mean, breaks=c(0,100,120,1000),right=FALSE)
complete.impbkdata$sbpcat<-relevel(complete.impbkdata$sbpcat,ref=2)
complete.impbkdata$dbpcat<-cut(complete.impbkdata$dbp_mean, breaks=c(0,70,85,1000),right=FALSE)
complete.impbkdata$dbpcat<-relevel(complete.impbkdata$dbpcat,ref=2)
complete.impbkdata$gluccat<-cut(complete.impbkdata$gluc, breaks=c(0,5,10,101),right=FALSE)
complete.impbkdata$ldlcat<-cut(complete.impbkdata$ldl, breaks=c(0,2.5,3.5,101),right=FALSE)
complete.impbkdata$ldlcat<-relevel(complete.impbkdata$ldlcat,ref=1)
complete.impbkdata$trigcat<-cut(complete.impbkdata$trigs, breaks=c(0,2.0,5.0,101),right=FALSE)
complete.impbkdata$hdlcat<-cut(complete.impbkdata$hdl, breaks=c(0,1.0,1.5,101),right=FALSE)
complete.impbkdata$hdlcat<-relevel(complete.impbkdata$hdlcat,ref=3)
impbkdata<-as.mids(complete.impbkdata)

bkdata$agecat<-cut(bkdata$age, breaks=c(0,40,50,60,70,80,150),right=FALSE)
bkdata$agecat<-relevel(bkdata$agecat,ref=3)
bkdata$irsadpercentcat<-cut(bkdata$irsadpercent, breaks=c(-1,40,80,101))
bkdata$irsadpercentcat<-relevel(bkdata$irsadpercentcat,ref=3)
bkdata$alcoholqtycat<-cut(bkdata$alcoholqty, breaks=c(-1,0,7,14,1500))
bkdata$bmicat<-cut(bkdata$BMI_trim, breaks=c(0,20,25,30,100),right=FALSE)
bkdata$bmicat<-relevel(bkdata$bmicat,ref=2)
bkdata$waistcat<-cut(bkdata$waist_trim, breaks=c(0,90,100,110,1000),right=FALSE)
bkdata$waistcat<-relevel(bkdata$waistcat,ref=1)
bkdata$whrcat<-cut(bkdata$WHR_trim, breaks=c(0,0.90,1.00,10),right=FALSE)
bkdata$whrcat<-relevel(bkdata$whrcat,ref=1)
bkdata$sbpcat<-cut(bkdata$sbp_mean, breaks=c(0,100,120,1000),right=FALSE)
bkdata$sbpcat<-relevel(bkdata$sbpcat,ref=2)
bkdata$dbpcat<-cut(bkdata$dbp_mean, breaks=c(0,70,85,1000),right=FALSE)
bkdata$dbpcat<-relevel(bkdata$dbpcat,ref=2)
bkdata$gluccat<-cut(bkdata$gluc, breaks=c(0,5,10,101),right=FALSE)
bkdata$ldlcat<-cut(bkdata$ldl, breaks=c(0,2.5,3.5,101),right=FALSE)
bkdata$ldlcat<-relevel(bkdata$ldlcat,ref=1)
bkdata$trigcat<-cut(bkdata$trigs, breaks=c(0,2.0,5.0,101),right=FALSE)
bkdata$hdlcat<-cut(bkdata$hdl, breaks=c(0,1.0,1.5,101),right=FALSE)
bkdata$hdlcat<-relevel(bkdata$hdlcat,ref=3)

crrall<-fit.mult.impute(Surv(timetohf,hfcomposite)~agecat+irsadpercentcat+alcoholqtycat+waistcat+bmicat+sex+smoker+hx_mi+hx_stroke+hx_ht+hx_chol+ hx_depression+hx_cabg+hx_stent+hx_arrhyth+hx_cad+hx_vd+hx_valve_dis+hx_kidney_dis+hx_osa+hx_asthma+hx_arthritis+hx_retinopathy +hx_migraine+hx_diab_any,fitter=crr,xtrans=impbkdata,data=bkdata);
crrallwhr<-fit.mult.impute(Surv(timetohf,hfcomposite)~agecat+irsadpercentcat+alcoholqtycat+whrcat+bmicat+sex+smoker+hx_mi+hx_stroke+hx_ht+hx_chol+ hx_depression+hx_cabg+hx_stent+hx_arrhyth+hx_cad+hx_vd+hx_valve_dis+hx_kidney_dis+hx_osa+hx_asthma+hx_arthritis+hx_retinopathy +hx_migraine+hx_diab_any,fitter=crr,xtrans=impbkdata,data=bkdata);
crrallsbp<-fit.mult.impute(Surv(timetohf,hfcomposite)~sbpcat+agecat+irsadpercentcat+alcoholqtycat+whrcat+bmicat+sex+smoker+hx_mi+hx_stroke+hx_ht+hx_chol+ hx_depression+hx_cabg+hx_stent+hx_arrhyth+hx_cad+hx_vd+hx_valve_dis+hx_kidney_dis+hx_osa+hx_asthma+hx_arthritis+hx_retinopathy +hx_migraine+hx_diab_any,fitter=crr,xtrans=impbkdata,data=bkdata);
crralldbp<-fit.mult.impute(Surv(timetohf,hfcomposite)~dbpcat+agecat+irsadpercentcat+alcoholqtycat+whrcat+bmicat+sex+smoker+hx_mi+hx_stroke+hx_ht+hx_chol+ hx_depression+hx_cabg+hx_stent+hx_arrhyth+hx_cad+hx_vd+hx_valve_dis+hx_kidney_dis+hx_osa+hx_asthma+hx_arthritis+hx_retinopathy +hx_migraine+hx_diab_any,fitter=crr,xtrans=impbkdata,data=bkdata);
crrallldl<-fit.mult.impute(Surv(timetohf,hfcomposite)~ldlcat+agecat+irsadpercentcat+alcoholqtycat+whrcat+bmicat+sex+smoker+hx_mi+hx_stroke+hx_ht+hx_chol+ hx_depression+hx_cabg+hx_stent+hx_arrhyth+hx_cad+hx_vd+hx_valve_dis+hx_kidney_dis+hx_osa+hx_asthma+hx_arthritis+hx_retinopathy +hx_migraine+hx_diab_any,fitter=crr,xtrans=impbkdata,data=bkdata);
crrallhdl<-fit.mult.impute(Surv(timetohf,hfcomposite)~hdlcat+agecat+irsadpercentcat+alcoholqtycat+whrcat+bmicat+sex+smoker+hx_mi+hx_stroke+hx_ht+hx_chol+ hx_depression+hx_cabg+hx_stent+hx_arrhyth+hx_cad+hx_vd+hx_valve_dis+hx_kidney_dis+hx_osa+hx_asthma+hx_arthritis+hx_retinopathy +hx_migraine+hx_diab_any,fitter=crr,xtrans=impbkdata,data=bkdata);
crralltrigs<-fit.mult.impute(Surv(timetohf,hfcomposite)~trigcat+agecat+irsadpercentcat+alcoholqtycat+whrcat+bmicat+sex+smoker+hx_mi+hx_stroke+hx_ht+hx_chol+ hx_depression+hx_cabg+hx_stent+hx_arrhyth+hx_cad+hx_vd+hx_valve_dis+hx_kidney_dis+hx_osa+hx_asthma+hx_arthritis+hx_retinopathy +hx_migraine+hx_diab_any,fitter=crr,xtrans=impbkdata,data=bkdata);
crrallgluc<-fit.mult.impute(Surv(timetohf,hfcomposite)~gluccat+agecat+irsadpercentcat+alcoholqtycat+whrcat+bmicat+sex+smoker+hx_mi+hx_stroke+hx_ht+hx_chol+ hx_depression+hx_cabg+hx_stent+hx_arrhyth+hx_cad+hx_vd+hx_valve_dis+hx_kidney_dis+hx_osa+hx_asthma+hx_arthritis+hx_retinopathy +hx_migraine+hx_diab_any,fitter=crr,xtrans=impbkdata,data=bkdata);

crrallascvd1<-fit.mult.impute(Surv(timetohf,hfcomposite)~agecat+irsadpercentcat+alcoholqtycat+waistcat+bmicat+sex+smoker+hx_ht+hx_chol+ hx_depression+hx_arrhyth+hx_valve_dis+hx_kidney_dis+hx_osa+hx_asthma+hx_arthritis+hx_retinopathy +hx_migraine+hx_diab_any+hx_mi+hx_cad+hx_cabg+hx_stent+hx_vd+hx_stroke,fitter=crr,xtrans=impbkdata,data=bkdata,subset=(bkdata$ascvd==1));
crrallwhrascvd1<-fit.mult.impute(Surv(timetohf,hfcomposite)~agecat+irsadpercentcat+alcoholqtycat+whrcat+bmicat+sex+smoker+hx_ht+hx_chol+ hx_depression+hx_arrhyth+hx_valve_dis+hx_kidney_dis+hx_osa+hx_asthma+hx_arthritis+hx_retinopathy +hx_migraine+hx_diab_any+hx_mi+hx_cad+hx_cabg+hx_stent+hx_vd+hx_stroke,fitter=crr,xtrans=impbkdata,data=bkdata,subset=(bkdata$ascvd==1));
crrallsbpascvd1<-fit.mult.impute(Surv(timetohf,hfcomposite)~sbpcat+agecat+irsadpercentcat+alcoholqtycat+whrcat+bmicat+sex+smoker+hx_ht+hx_chol+ hx_depression+hx_arrhyth+hx_valve_dis+hx_kidney_dis+hx_osa+hx_asthma+hx_arthritis+hx_retinopathy +hx_migraine+hx_diab_any+hx_mi+hx_cad+hx_cabg+hx_stent+hx_vd+hx_stroke,fitter=crr,xtrans=impbkdata,data=bkdata,subset=(bkdata$ascvd==1));
crralldbpascvd1<-fit.mult.impute(Surv(timetohf,hfcomposite)~dbpcat+agecat+irsadpercentcat+alcoholqtycat+whrcat+bmicat+sex+smoker+hx_ht+hx_chol+ hx_depression+hx_arrhyth+hx_valve_dis+hx_kidney_dis+hx_osa+hx_asthma+hx_arthritis+hx_retinopathy +hx_migraine+hx_diab_any+hx_mi+hx_cad+hx_cabg+hx_stent+hx_vd+hx_stroke,fitter=crr,xtrans=impbkdata,data=bkdata,subset=(bkdata$ascvd==1));
crrallldlascvd1<-fit.mult.impute(Surv(timetohf,hfcomposite)~ldlcat+agecat+irsadpercentcat+alcoholqtycat+whrcat+bmicat+sex+smoker+hx_ht+hx_chol+ hx_depression+hx_arrhyth+hx_valve_dis+hx_kidney_dis+hx_osa+hx_asthma+hx_arthritis+hx_retinopathy +hx_migraine+hx_diab_any+hx_mi+hx_cad+hx_cabg+hx_stent+hx_vd+hx_stroke,fitter=crr,xtrans=impbkdata,data=bkdata,subset=(bkdata$ascvd==1));
crrallhdlascvd1<-fit.mult.impute(Surv(timetohf,hfcomposite)~hdlcat+agecat+irsadpercentcat+alcoholqtycat+whrcat+bmicat+sex+smoker+hx_ht+hx_chol+ hx_depression+hx_arrhyth+hx_valve_dis+hx_kidney_dis+hx_osa+hx_asthma+hx_arthritis+hx_retinopathy +hx_migraine+hx_diab_any+hx_mi+hx_cad+hx_cabg+hx_stent+hx_vd+hx_stroke,fitter=crr,xtrans=impbkdata,data=bkdata,subset=(bkdata$ascvd==1));
crralltrigsascvd1<-fit.mult.impute(Surv(timetohf,hfcomposite)~trigcat+agecat+irsadpercentcat+alcoholqtycat+whrcat+bmicat+sex+smoker+hx_ht+hx_chol+ hx_depression+hx_arrhyth+hx_valve_dis+hx_kidney_dis+hx_osa+hx_asthma+hx_arthritis+hx_retinopathy +hx_migraine+hx_diab_any+hx_mi+hx_cad+hx_cabg+hx_stent+hx_vd+hx_stroke,fitter=crr,xtrans=impbkdata,data=bkdata,subset=(bkdata$ascvd==1));
crrallglucascvd1<-fit.mult.impute(Surv(timetohf,hfcomposite)~gluccat+agecat+irsadpercentcat+alcoholqtycat+whrcat+bmicat+sex+smoker+hx_ht+hx_chol+ hx_depression+hx_arrhyth+hx_valve_dis+hx_kidney_dis+hx_osa+hx_asthma+hx_arthritis+hx_retinopathy +hx_migraine+hx_diab_any+hx_mi+hx_cad+hx_cabg+hx_stent+hx_vd+hx_stroke,fitter=crr,xtrans=impbkdata,data=bkdata,subset=(bkdata$ascvd==1));

crrallascvd0<-fit.mult.impute(Surv(timetohf,hfcomposite)~agecat+irsadpercentcat+alcoholqtycat+waistcat+bmicat+sex+smoker+hx_ht+hx_chol+ hx_depression+hx_arrhyth+hx_valve_dis+hx_kidney_dis+hx_osa+hx_asthma+hx_arthritis+hx_retinopathy +hx_migraine+hx_diab_any,fitter=crr,xtrans=impbkdata,data=bkdata,subset=(bkdata$ascvd==0));
crrallwhrascvd0<-fit.mult.impute(Surv(timetohf,hfcomposite)~agecat+irsadpercentcat+alcoholqtycat+whrcat+bmicat+sex+smoker+hx_ht+hx_chol+ hx_depression+hx_arrhyth+hx_valve_dis+hx_kidney_dis+hx_osa+hx_asthma+hx_arthritis+hx_retinopathy +hx_migraine+hx_diab_any,fitter=crr,xtrans=impbkdata,data=bkdata,subset=(bkdata$ascvd==0));
crrallsbpascvd0<-fit.mult.impute(Surv(timetohf,hfcomposite)~sbpcat+agecat+irsadpercentcat+alcoholqtycat+whrcat+bmicat+sex+smoker+hx_ht+hx_chol+ hx_depression+hx_arrhyth+hx_valve_dis+hx_kidney_dis+hx_osa+hx_asthma+hx_arthritis+hx_retinopathy +hx_migraine+hx_diab_any,fitter=crr,xtrans=impbkdata,data=bkdata,subset=(bkdata$ascvd==0));
crralldbpascvd0<-fit.mult.impute(Surv(timetohf,hfcomposite)~dbpcat+agecat+irsadpercentcat+alcoholqtycat+whrcat+bmicat+sex+smoker+hx_ht+hx_chol+ hx_depression+hx_arrhyth+hx_valve_dis+hx_kidney_dis+hx_osa+hx_asthma+hx_arthritis+hx_retinopathy +hx_migraine+hx_diab_any,fitter=crr,xtrans=impbkdata,data=bkdata,subset=(bkdata$ascvd==0));
crrallldlascvd0<-fit.mult.impute(Surv(timetohf,hfcomposite)~ldlcat+agecat+irsadpercentcat+alcoholqtycat+whrcat+bmicat+sex+smoker+hx_ht+hx_chol+ hx_depression+hx_arrhyth+hx_valve_dis+hx_kidney_dis+hx_osa+hx_asthma+hx_arthritis+hx_retinopathy +hx_migraine+hx_diab_any,fitter=crr,xtrans=impbkdata,data=bkdata,subset=(bkdata$ascvd==0));
crrallhdlascvd0<-fit.mult.impute(Surv(timetohf,hfcomposite)~hdlcat+agecat+irsadpercentcat+alcoholqtycat+whrcat+bmicat+sex+smoker+hx_ht+hx_chol+ hx_depression+hx_arrhyth+hx_valve_dis+hx_kidney_dis+hx_osa+hx_asthma+hx_arthritis+hx_retinopathy +hx_migraine+hx_diab_any,fitter=crr,xtrans=impbkdata,data=bkdata,subset=(bkdata$ascvd==0));
crralltrigsascvd0<-fit.mult.impute(Surv(timetohf,hfcomposite)~trigcat+agecat+irsadpercentcat+alcoholqtycat+whrcat+bmicat+sex+smoker+hx_ht+hx_chol+ hx_depression+hx_arrhyth+hx_valve_dis+hx_kidney_dis+hx_osa+hx_asthma+hx_arthritis+hx_retinopathy +hx_migraine+hx_diab_any,fitter=crr,xtrans=impbkdata,data=bkdata,subset=(bkdata$ascvd==0));
crrallglucascvd0<-fit.mult.impute(Surv(timetohf,hfcomposite)~gluccat+agecat+irsadpercentcat+alcoholqtycat+whrcat+bmicat+sex+smoker+hx_ht+hx_chol+ hx_depression+hx_arrhyth+hx_valve_dis+hx_kidney_dis+hx_osa+hx_asthma+hx_arthritis+hx_retinopathy +hx_migraine+hx_diab_any,fitter=crr,xtrans=impbkdata,data=bkdata,subset=(bkdata$ascvd==0));

crrall
crrallwhr
crrallsbp
crralldbp
crrallldl
crrallhdl
crralltrigs
crrallgluc

crrallascvd1
crrallwhrascvd1
crrallsbpascvd1
crralldbpascvd1
crrallldlascvd1
crrallhdlascvd1
crralltrigsascvd1
crrallglucascvd1

crrallascvd0
crrallwhrascvd0
crrallsbpascvd0
crralldbpascvd0
crrallldlascvd0
crrallhdlascvd0
crralltrigsascvd0
crrallglucascvd0

crrmorb<-fit.mult.impute(Surv(timetohf,hfcomposite)~rcs(age,3)+rcs(irsadpercent,3)+rcs(alcoholqty,3)+rcs(waist_trim,4)+rcs(BMI_trim,3)+sex+morbcount,fitter=crr,xtrans=impbkdata,data=bkdata);
crrmorbascvd1<-fit.mult.impute(Surv(timetohf,hfcomposite)~rcs(age,3)+rcs(irsadpercent,3)+rcs(alcoholqty,3)+rcs(waist_trim,4)+rcs(BMI_trim,3)+sex+morbcount,fitter=crr,xtrans=impbkdata,data=bkdata,subset=(bkdata$ascvd==1));
crrmorbascvd0<-fit.mult.impute(Surv(timetohf,hfcomposite)~rcs(age,3)+rcs(irsadpercent,3)+rcs(alcoholqty,3)+rcs(waist_trim,4)+rcs(BMI_trim,3)+sex+morbcount,fitter=crr,xtrans=impbkdata,data=bkdata,subset=(bkdata$ascvd==0));
crrmorb
crrmorbascvd1
crrmorbascvd0

#Sensitivity analysis subdistribution models using complete case to manage missing data

crrcompleteall<-crr(Surv(timetohf,hfcomposite)~agecat+irsadpercentcat+alcoholqtycat+waistcat+bmicat+sex+smoker+hx_mi+hx_stroke+hx_ht+hx_chol+ hx_depression+hx_cabg+hx_stent+hx_arrhyth+hx_cad+hx_vd+hx_valve_dis+hx_kidney_dis+hx_osa+hx_asthma+hx_arthritis+hx_retinopathy +hx_migraine+hx_diab_any,data=bkdata);
crrcompleteallwhr<-crr(Surv(timetohf,hfcomposite)~agecat+irsadpercentcat+alcoholqtycat+whrcat+bmicat+sex+smoker+hx_mi+hx_stroke+hx_ht+hx_chol+ hx_depression+hx_cabg+hx_stent+hx_arrhyth+hx_cad+hx_vd+hx_valve_dis+hx_kidney_dis+hx_osa+hx_asthma+hx_arthritis+hx_retinopathy +hx_migraine+hx_diab_any,data=bkdata);
crrcompleteallsbp<-crr(Surv(timetohf,hfcomposite)~sbpcat+agecat+irsadpercentcat+alcoholqtycat+whrcat+bmicat+sex+smoker+hx_mi+hx_stroke+hx_ht+hx_chol+ hx_depression+hx_cabg+hx_stent+hx_arrhyth+hx_cad+hx_vd+hx_valve_dis+hx_kidney_dis+hx_osa+hx_asthma+hx_arthritis+hx_retinopathy +hx_migraine+hx_diab_any,data=bkdata);
crrcompletealldbp<-crr(Surv(timetohf,hfcomposite)~dbpcat+agecat+irsadpercentcat+alcoholqtycat+whrcat+bmicat+sex+smoker+hx_mi+hx_stroke+hx_ht+hx_chol+ hx_depression+hx_cabg+hx_stent+hx_arrhyth+hx_cad+hx_vd+hx_valve_dis+hx_kidney_dis+hx_osa+hx_asthma+hx_arthritis+hx_retinopathy +hx_migraine+hx_diab_any,data=bkdata);
crrcompleteallldl<-crr(Surv(timetohf,hfcomposite)~ldlcat+agecat+irsadpercentcat+alcoholqtycat+whrcat+bmicat+sex+smoker+hx_mi+hx_stroke+hx_ht+hx_chol+ hx_depression+hx_cabg+hx_stent+hx_arrhyth+hx_cad+hx_vd+hx_valve_dis+hx_kidney_dis+hx_osa+hx_asthma+hx_arthritis+hx_retinopathy +hx_migraine+hx_diab_any,data=bkdata);
crrcompleteallhdl<-crr(Surv(timetohf,hfcomposite)~hdlcat+agecat+irsadpercentcat+alcoholqtycat+whrcat+bmicat+sex+smoker+hx_mi+hx_stroke+hx_ht+hx_chol+ hx_depression+hx_cabg+hx_stent+hx_arrhyth+hx_cad+hx_vd+hx_valve_dis+hx_kidney_dis+hx_osa+hx_asthma+hx_arthritis+hx_retinopathy +hx_migraine+hx_diab_any,data=bkdata);
crrcompletealltrigs<-crr(Surv(timetohf,hfcomposite)~trigcat+agecat+irsadpercentcat+alcoholqtycat+whrcat+bmicat+sex+smoker+hx_mi+hx_stroke+hx_ht+hx_chol+ hx_depression+hx_cabg+hx_stent+hx_arrhyth+hx_cad+hx_vd+hx_valve_dis+hx_kidney_dis+hx_osa+hx_asthma+hx_arthritis+hx_retinopathy +hx_migraine+hx_diab_any,data=bkdata);
crrcompleteallgluc<-crr(Surv(timetohf,hfcomposite)~gluccat+agecat+irsadpercentcat+alcoholqtycat+whrcat+bmicat+sex+smoker+hx_mi+hx_stroke+hx_ht+hx_chol+ hx_depression+hx_cabg+hx_stent+hx_arrhyth+hx_cad+hx_vd+hx_valve_dis+hx_kidney_dis+hx_osa+hx_asthma+hx_arthritis+hx_retinopathy +hx_migraine+hx_diab_any,data=bkdata);

crrcompleteallascvd1<-crr(Surv(timetohf,hfcomposite)~agecat+irsadpercentcat+alcoholqtycat+waistcat+bmicat+sex+smoker+hx_ht+hx_chol+ hx_depression+hx_arrhyth+hx_valve_dis+hx_kidney_dis+hx_osa+hx_asthma+hx_arthritis+hx_retinopathy +hx_migraine+hx_diab_any+hx_mi+hx_cad+hx_cabg+hx_stent+hx_vd+hx_stroke,data=subset(bkdata, ascvd == 1));
crrcompleteallwhrascvd1<-crr(Surv(timetohf,hfcomposite)~agecat+irsadpercentcat+alcoholqtycat+whrcat+bmicat+sex+smoker+hx_ht+hx_chol+ hx_depression+hx_arrhyth+hx_valve_dis+hx_kidney_dis+hx_osa+hx_asthma+hx_arthritis+hx_retinopathy +hx_migraine+hx_diab_any+hx_mi+hx_cad+hx_cabg+hx_stent+hx_vd+hx_stroke,data=subset(bkdata, ascvd == 1));
crrcompleteallsbpascvd1<-crr(Surv(timetohf,hfcomposite)~sbpcat+agecat+irsadpercentcat+alcoholqtycat+whrcat+bmicat+sex+smoker+hx_ht+hx_chol+ hx_depression+hx_arrhyth+hx_valve_dis+hx_kidney_dis+hx_osa+hx_asthma+hx_arthritis+hx_retinopathy +hx_migraine+hx_diab_any+hx_mi+hx_cad+hx_cabg+hx_stent+hx_vd+hx_stroke,data=subset(bkdata, ascvd == 1));
crrcompletealldbpascvd1<-crr(Surv(timetohf,hfcomposite)~dbpcat+agecat+irsadpercentcat+alcoholqtycat+whrcat+bmicat+sex+smoker+hx_ht+hx_chol+ hx_depression+hx_arrhyth+hx_valve_dis+hx_kidney_dis+hx_osa+hx_asthma+hx_arthritis+hx_retinopathy +hx_migraine+hx_diab_any+hx_mi+hx_cad+hx_cabg+hx_stent+hx_vd+hx_stroke,data=subset(bkdata, ascvd == 1));
crrcompleteallldlascvd1<-crr(Surv(timetohf,hfcomposite)~ldlcat+agecat+irsadpercentcat+alcoholqtycat+whrcat+bmicat+sex+smoker+hx_ht+hx_chol+ hx_depression+hx_arrhyth+hx_valve_dis+hx_kidney_dis+hx_osa+hx_asthma+hx_arthritis+hx_retinopathy +hx_migraine+hx_diab_any+hx_mi+hx_cad+hx_cabg+hx_stent+hx_vd+hx_stroke,data=subset(bkdata, ascvd == 1));
crrcompleteallhdlascvd1<-crr(Surv(timetohf,hfcomposite)~hdlcat+agecat+irsadpercentcat+alcoholqtycat+whrcat+bmicat+sex+smoker+hx_ht+hx_chol+ hx_depression+hx_arrhyth+hx_valve_dis+hx_kidney_dis+hx_osa+hx_asthma+hx_arthritis+hx_retinopathy +hx_migraine+hx_diab_any+hx_mi+hx_cad+hx_cabg+hx_stent+hx_vd+hx_stroke,data=subset(bkdata, ascvd == 1));
crrcompletealltrigsascvd1<-crr(Surv(timetohf,hfcomposite)~trigcat+agecat+irsadpercentcat+alcoholqtycat+whrcat+bmicat+sex+smoker+hx_ht+hx_chol+ hx_depression+hx_arrhyth+hx_valve_dis+hx_kidney_dis+hx_osa+hx_asthma+hx_arthritis+hx_retinopathy +hx_migraine+hx_diab_any+hx_mi+hx_cad+hx_cabg+hx_stent+hx_vd+hx_stroke,data=subset(bkdata, ascvd == 1));
crrcompleteallglucascvd1<-crr(Surv(timetohf,hfcomposite)~gluccat+agecat+irsadpercentcat+alcoholqtycat+whrcat+bmicat+sex+smoker+hx_ht+hx_chol+ hx_depression+hx_arrhyth+hx_valve_dis+hx_kidney_dis+hx_osa+hx_asthma+hx_arthritis+hx_retinopathy +hx_migraine+hx_diab_any+hx_mi+hx_cad+hx_cabg+hx_stent+hx_vd+hx_stroke,data=subset(bkdata, ascvd == 1));

crrcompleteallascvd0<-crr(Surv(timetohf,hfcomposite)~agecat+irsadpercentcat+alcoholqtycat+waistcat+bmicat+sex+smoker+hx_ht+hx_chol+ hx_depression+hx_arrhyth+hx_valve_dis+hx_kidney_dis+hx_osa+hx_asthma+hx_arthritis+hx_retinopathy +hx_migraine+hx_diab_any,data=subset(bkdata, ascvd == 0));
crrcompleteallwhrascvd0<-crr(Surv(timetohf,hfcomposite)~agecat+irsadpercentcat+alcoholqtycat+whrcat+bmicat+sex+smoker+hx_ht+hx_chol+ hx_depression+hx_arrhyth+hx_valve_dis+hx_kidney_dis+hx_osa+hx_asthma+hx_arthritis+hx_retinopathy +hx_migraine+hx_diab_any,data=subset(bkdata, ascvd == 0));
crrcompleteallsbpascvd0<-crr(Surv(timetohf,hfcomposite)~sbpcat+agecat+irsadpercentcat+alcoholqtycat+whrcat+bmicat+sex+smoker+hx_ht+hx_chol+ hx_depression+hx_arrhyth+hx_valve_dis+hx_kidney_dis+hx_osa+hx_asthma+hx_arthritis+hx_retinopathy +hx_migraine+hx_diab_any,data=subset(bkdata, ascvd == 0));
crrcompletealldbpascvd0<-crr(Surv(timetohf,hfcomposite)~dbpcat+agecat+irsadpercentcat+alcoholqtycat+whrcat+bmicat+sex+smoker+hx_ht+hx_chol+ hx_depression+hx_arrhyth+hx_valve_dis+hx_kidney_dis+hx_osa+hx_asthma+hx_arthritis+hx_retinopathy +hx_migraine+hx_diab_any,data=subset(bkdata, ascvd == 0));
crrcompleteallldlascvd0<-crr(Surv(timetohf,hfcomposite)~ldlcat+agecat+irsadpercentcat+alcoholqtycat+whrcat+bmicat+sex+smoker+hx_ht+hx_chol+ hx_depression+hx_arrhyth+hx_valve_dis+hx_kidney_dis+hx_osa+hx_asthma+hx_arthritis+hx_retinopathy +hx_migraine+hx_diab_any,data=subset(bkdata, ascvd == 0));
crrcompleteallhdlascvd0<-crr(Surv(timetohf,hfcomposite)~hdlcat+agecat+irsadpercentcat+alcoholqtycat+whrcat+bmicat+sex+smoker+hx_ht+hx_chol+ hx_depression+hx_arrhyth+hx_valve_dis+hx_kidney_dis+hx_osa+hx_asthma+hx_arthritis+hx_retinopathy +hx_migraine+hx_diab_any,data=subset(bkdata, ascvd == 0));
crrcompletealltrigsascvd0<-crr(Surv(timetohf,hfcomposite)~trigcat+agecat+irsadpercentcat+alcoholqtycat+whrcat+bmicat+sex+smoker+hx_ht+hx_chol+ hx_depression+hx_arrhyth+hx_valve_dis+hx_kidney_dis+hx_osa+hx_asthma+hx_arthritis+hx_retinopathy +hx_migraine+hx_diab_any,data=subset(bkdata, ascvd == 0));
crrcompleteallglucascvd0<-crr(Surv(timetohf,hfcomposite)~gluccat+agecat+irsadpercentcat+alcoholqtycat+whrcat+bmicat+sex+smoker+hx_ht+hx_chol+ hx_depression+hx_arrhyth+hx_valve_dis+hx_kidney_dis+hx_osa+hx_asthma+hx_arthritis+hx_retinopathy +hx_migraine+hx_diab_any,data=subset(bkdata, ascvd == 0));

crrcompleteall
crrcompleteallwhr
crrcompleteallsbp
crrcompletealldbp
crrcompleteallldl
crrcompleteallhdl
crrcompletealltrigs
crrcompleteallgluc

crrcompleteallascvd1
crrcompleteallwhrascvd1
crrcompleteallsbpascvd1
crrcompletealldbpascvd1
crrcompleteallldlascvd1
crrcompleteallhdlascvd1
crrcompletealltrigsascvd1
crrcompleteallglucascvd1

crrcompleteallascvd0
crrcompleteallwhrascvd0
crrcompleteallsbpascvd0
crrcompletealldbpascvd0
crrcompleteallldlascvd0
crrcompleteallhdlascvd0
crrcompletealltrigsascvd0
crrcompleteallglucascvd0

crrcompletemorb<-crr(Surv(timetohf,hfcomposite)~rcs(age,3)+rcs(irsadpercent,3)+rcs(alcoholqty,3)+rcs(waist_trim,4)+rcs(BMI_trim,3)+sex+morbcount,data=bkdata);
crrcompletemorbascvd1<-crr(Surv(timetohf,hfcomposite)~rcs(age,3)+rcs(irsadpercent,3)+rcs(alcoholqty,3)+rcs(waist_trim,4)+rcs(BMI_trim,3)+sex+morbcount,data=subset(bkdata, ascvd == 1));
crrcompletemorbascvd0<-crr(Surv(timetohf,hfcomposite)~rcs(age,3)+rcs(irsadpercent,3)+rcs(alcoholqty,3)+rcs(waist_trim,4)+rcs(BMI_trim,3)+sex+morbcount,data=subset(bkdata, ascvd == 0));
crrcompletemorb
crrcompletemorbascvd1
crrcompletemorbascvd0

#Cox regressions for Figure 4, each model tested with 3,4,5 knots for spline terms and no. of knots selected based on minimum AIC

#age spline

coxmage<- fit.mult.impute(Surv(timetohf,hfany)~rcs(age,3)+rcs(irsadpercent,3)+rcs(alcoholqty,3)+rcs(waist_trim,4)+rcs(BMI_trim,3)+sex+smoker+hx_mi+hx_stroke+hx_ht+hx_chol+ hx_depression+hx_cabg+hx_stent+hx_arrhyth+hx_cad+hx_vd+hx_valve_dis+hx_kidney_dis+hx_osa+hx_asthma+hx_arthritis+hx_retinopathy +hx_migraine+hx_diab_any,fitter=cph,xtrans=impbkdata,data=bkdata)
coxmagelinear<- fit.mult.impute(Surv(timetohf,hfany)~age+rcs(irsadpercent,3)+rcs(alcoholqty,3)+rcs(waist_trim,4)+rcs(BMI_trim,3)+sex+smoker+hx_mi+hx_stroke+hx_ht+hx_chol+ hx_depression+hx_cabg+hx_stent+hx_arrhyth+hx_cad+hx_vd+hx_valve_dis+hx_kidney_dis+hx_osa+hx_asthma+hx_arthritis+hx_retinopathy +hx_migraine+hx_diab_any,fitter=cph,xtrans=impbkdata,data=bkdata)
coxmageno<- fit.mult.impute(Surv(timetohf,hfany)~rcs(alcoholqty,3)+rcs(irsadpercent,3)+ rcs(waist_trim,4)+rcs(BMI_trim,3)+sex+smoker+hx_mi+hx_stroke+hx_ht+hx_chol+ hx_depression+hx_cabg+hx_stent+hx_arrhyth+hx_cad+hx_vd+hx_valve_dis+hx_kidney_dis+hx_osa+hx_asthma+hx_arthritis+hx_retinopathy+hx_migraine+hx_diab_any,fitter=cph,xtrans=impbkdata,data=bkdata)
AIC(coxmage)
lrtest(coxmage,coxmageno)
lrtest(coxmage,coxmagelinear)
prcoxage<-Predict(coxmage,age=seq(30,85,by=1),ref.zero=TRUE,fun=exp)
ggage<-ggplot(data=prcoxage,colfill='dodgerblue2',xlab="Age",ylab="HR (95%CI)",adj.subtitle=FALSE)+geom_hline(aes(yintercept=1),linetype=3,col="black",size=.4)+geom_line(aes(x=age,y=yhat),col='dodgerblue2',size=.75);
ggage$coordinates$limits$y <- NULL;
ggage+ scale_y_continuous(trans='log2',limits=c(0.25,18),breaks=c(0.5,1,2,4,8,16))+theme_classic(base_size=13)+ annotate("text",label="P-overall < 0.001",x=40,y=16,colour="black",size=3.5)+ annotate("text",label="P-nonlinear < 0.001",x=40,y=12,colour="black",size=3.5)

#ses spline

coxmirsad<- fit.mult.impute(Surv(timetohf,hfany)~rcs(age,3)+rcs(irsadpercent,3)+rcs(alcoholqty,3)+rcs(waist_trim,4)+rcs(BMI_trim,3)+sex+smoker+hx_mi+hx_stroke+hx_ht+hx_chol+ hx_depression+hx_cabg+hx_stent+hx_arrhyth+hx_cad+hx_vd+hx_valve_dis+hx_kidney_dis+hx_osa+hx_asthma+hx_arthritis+hx_retinopathy +hx_migraine+hx_diab_any,fitter=cph,xtrans=impbkdata,data=bkdata)
coxmirsadlinear<- fit.mult.impute(Surv(timetohf,hfany)~rcs(age,3)+irsadpercent+rcs(alcoholqty,3)+rcs(waist_trim,4)+rcs(BMI_trim,3)+sex+smoker+hx_mi+hx_stroke+hx_ht+hx_chol+ hx_depression+hx_cabg+hx_stent+hx_arrhyth+hx_cad+hx_vd+hx_valve_dis+hx_kidney_dis+hx_osa+hx_asthma+hx_arthritis+hx_retinopathy +hx_migraine+hx_diab_any,fitter=cph,xtrans=impbkdata,data=bkdata)
coxmirsadno<- fit.mult.impute(Surv(timetohf,hfany)~rcs(age,3)+ rcs(alcoholqty,3)+rcs(waist_trim,4)+rcs(BMI_trim,3)+sex+smoker+hx_mi+hx_stroke+hx_ht+hx_chol+ hx_depression+hx_cabg+hx_stent+hx_arrhyth+hx_cad+hx_vd+hx_valve_dis+hx_kidney_dis+hx_osa+hx_asthma+hx_arthritis+hx_retinopathy+hx_migraine+hx_diab_any,fitter=cph,xtrans=impbkdata,data=bkdata)
AIC(coxmirsad)
lrtest(coxmirsad,coxmirsadno)
lrtest(coxmirsad,coxmirsadlinear)
prcoxirsad<-Predict(coxmirsad,irsadpercent=seq(0,100,by=1),ref.zero=TRUE,fun=exp)
ggirsad<-ggplot(data=prcoxirsad,colfill='dodgerblue2',xlab="Socioeconomic Status",ylab="HR (95%CI)",adj.subtitle=FALSE)+geom_hline(aes(yintercept=1),linetype=3,col="black",size=.4)+geom_line(aes(x=irsadpercent,y=yhat),col='dodgerblue2',size=.75);
ggirsad$coordinates$limits$y <- NULL;
ggirsad+ scale_y_continuous(trans='log2',limits=c(0.3,5),breaks=c(0.5,1,2,4))+theme_classic(base_size=13)+ annotate("text",label="P-overall < 0.001",x=20,y=4.5,colour="black",size=3.5)+ annotate("text",label="P-nonlinear = 0.044",x=20,y=3.5,colour="black",size=3.5)


#alcohol spline

dd<-datadist(bkdata)
options(datadist="dd")
coxmalcoholqty<- fit.mult.impute(Surv(timetohf,hfany)~rcs(age,3)+rcs(irsadpercent,3)+rcs(alcoholqty,3)+rcs(waist_trim,4)+rcs(BMI_trim,3)+sex+smoker+hx_mi+hx_stroke+hx_ht+hx_chol+ hx_depression+hx_cabg+hx_stent+hx_arrhyth+hx_cad+hx_vd+hx_valve_dis+hx_kidney_dis+hx_osa+hx_asthma+hx_arthritis+hx_retinopathy +hx_migraine+hx_diab_any,fitter=cph,xtrans=impbkdata,data=bkdata)
coxmalcoholqtylinear<- fit.mult.impute(Surv(timetohf,hfany)~rcs(age,3)+rcs(irsadpercent,3)+alcoholqty+rcs(waist_trim,4)+rcs(BMI_trim,3)+sex+smoker+hx_mi+hx_stroke+hx_ht+hx_chol+ hx_depression+hx_cabg+hx_stent+hx_arrhyth+hx_cad+hx_vd+hx_valve_dis+hx_kidney_dis+hx_osa+hx_asthma+hx_arthritis+hx_retinopathy +hx_migraine+hx_diab_any,fitter=cph,xtrans=impbkdata,data=bkdata)
coxmalcoholqtyno<- fit.mult.impute(Surv(timetohf,hfany)~rcs(age,3)+rcs(irsadpercent,3)+ rcs(waist_trim,4)+rcs(BMI_trim,3)+sex+smoker+hx_mi+hx_stroke+hx_ht+hx_chol+ hx_depression+hx_cabg+hx_stent+hx_arrhyth+hx_cad+hx_vd+hx_valve_dis+hx_kidney_dis+hx_osa+hx_asthma+hx_arthritis+hx_retinopathy+hx_migraine+hx_diab_any,fitter=cph,xtrans=impbkdata,data=bkdata)
AIC(coxmalcoholqty)
lrtest(coxmalcoholqty,coxmalcoholqtyno)
lrtest(coxmalcoholqty,coxmalcoholqtylinear)
prcoxalcoholqty<-Predict(coxmalcoholqty,alcoholqty=seq(0,42,by=1),ref.zero=TRUE,fun=exp)
ggalcoholqty<-ggplot(data=prcoxalcoholqty,colfill='dodgerblue2',xlab="Alcohol (SD/week)",ylab="HR (95%CI)",adj.subtitle=FALSE)+geom_hline(aes(yintercept=1),linetype=3,col="black",size=.4)+geom_line(aes(x=alcoholqty,y=yhat),col='dodgerblue2',size=.75);
ggalcoholqty$coordinates$limits$y <- NULL;
ggalcoholqty+ scale_y_continuous(trans='log2',limits=c(0.3,5),breaks=c(0.5,1,2,4))+theme_classic(base_size=13)+ annotate("text",label="P-overall < 0.001",x=10,y=4.5,colour="black",size=3.5)+ annotate("text",label="P-nonlinear < 0.001",x=10,y=3.5,colour="black",size=3.5)

#bmi spline

coxmbmi<- fit.mult.impute(Surv(timetohf,hfany)~rcs(age,3)+rcs(irsadpercent,3)+rcs(alcoholqty,3)+ rcs(BMI_trim,3)+sex+smoker+hx_mi+hx_stroke+hx_ht+hx_chol+ hx_depression+hx_cabg+hx_stent+hx_arrhyth+hx_cad+hx_vd+hx_valve_dis+hx_kidney_dis+hx_osa+hx_asthma+hx_arthritis+hx_retinopathy +hx_migraine+hx_diab_any,fitter=cph,xtrans=impbkdata,data=bkdata)
coxmbmilinear<- fit.mult.impute(Surv(timetohf,hfany)~rcs(age,3)+rcs(irsadpercent,3)+rcs(alcoholqty,3)+ BMI_trim+sex+smoker+hx_mi+hx_stroke+hx_ht+hx_chol+ hx_depression+hx_cabg+hx_stent+hx_arrhyth+hx_cad+hx_vd+hx_valve_dis+hx_kidney_dis+hx_osa+hx_asthma+hx_arthritis+hx_retinopathy +hx_migraine+hx_diab_any,fitter=cph,xtrans=impbkdata,data=bkdata)
coxmbmino<- fit.mult.impute(Surv(timetohf,hfany)~rcs(age,3)+rcs(irsadpercent,3)+rcs(alcoholqty,3)+ sex+smoker+hx_mi+hx_stroke+hx_ht+hx_chol+ hx_depression+hx_cabg+hx_stent+hx_arrhyth+hx_cad+hx_vd+hx_valve_dis+hx_kidney_dis+hx_osa+hx_asthma+hx_arthritis+hx_retinopathy +hx_migraine+hx_diab_any,fitter=cph,xtrans=impbkdata,data=bkdata)
AIC(coxmbmi)
lrtest(coxmbmi,coxmbmino)
lrtest(coxmbmi,coxmbmilinear)
prcoxbmi<-Predict(coxmbmi,BMI_trim=seq(18,40,by=.1),ref.zero=TRUE,fun=exp)
ggbmi<-ggplot(data=prcoxbmi,colfill='dodgerblue2',xlab="Body Mass Index",ylab="HR (95%CI)",adj.subtitle=FALSE)+geom_hline(aes(yintercept=1),linetype=3,col="black",size=.4)+geom_line(aes(x=BMI_trim,y=yhat),col='dodgerblue2',size=.75);
ggbmi$coordinates$limits$y <- NULL;
ggbmi+ scale_y_continuous(trans='log2',limits=c(0.3,5),breaks=c(0.5,1,2,4))+theme_classic(base_size=13)+ annotate("text",label="P-overall < 0.001",x=24,y=4.5,colour="black",size=3.5)+ annotate("text",label="P-nonlinear < 0.001",x=24,y=3.5,colour="black",size=3.5)

#waist spline

coxmwaist<- fit.mult.impute(Surv(timetohf,hfany)~rcs(age,3)+rcs(irsadpercent,3)+rcs(alcoholqty,3)+ rcs(waist_trim,4)+sex+smoker+hx_mi+hx_stroke+hx_ht+hx_chol+ hx_depression+hx_cabg+hx_stent+hx_arrhyth+hx_cad+hx_vd+hx_valve_dis+hx_kidney_dis+hx_osa+hx_asthma+hx_arthritis+hx_retinopathy +hx_migraine+hx_diab_any,fitter=cph,xtrans=impbkdata,data=bkdata)
coxmwaistlinear<- fit.mult.impute(Surv(timetohf,hfany)~rcs(age,3)+rcs(irsadpercent,3)+rcs(alcoholqty,3)+ waist_trim+sex+smoker+hx_mi+hx_stroke+hx_ht+hx_chol+ hx_depression+hx_cabg+hx_stent+hx_arrhyth+hx_cad+hx_vd+hx_valve_dis+hx_kidney_dis+hx_osa+hx_asthma+hx_arthritis+hx_retinopathy +hx_migraine+hx_diab_any,fitter=cph,xtrans=impbkdata,data=bkdata)
coxmwaistno<- fit.mult.impute(Surv(timetohf,hfany)~rcs(age,3)+rcs(irsadpercent,3)+rcs(alcoholqty,3)+ sex+smoker+hx_mi+hx_stroke+hx_ht+hx_chol+ hx_depression+hx_cabg+hx_stent+hx_arrhyth+hx_cad+hx_vd+hx_valve_dis+hx_kidney_dis+hx_osa+hx_asthma+hx_arthritis+hx_retinopathy +hx_migraine+hx_diab_any,fitter=cph,xtrans=impbkdata,data=bkdata)
AIC(coxmwaist)
lrtest(coxmwaist,coxmwaistno)
lrtest(coxmwaist,coxmwaistlinear)
prcoxwaist<-Predict(coxmwaist,waist_trim=seq(70,130,by=.1),ref.zero=TRUE,fun=exp)
ggwaist<-ggplot(data=prcoxwaist,colfill='dodgerblue2',xlab="Waist Circumference",ylab="HR (95%CI)",adj.subtitle=FALSE)+geom_hline(aes(yintercept=1),linetype=3,col="black",size=.4)+geom_line(aes(x=waist_trim,y=yhat),col='dodgerblue2',size=.75);
ggwaist$coordinates$limits$y <- NULL;
ggwaist+ scale_y_continuous(trans='log2',limits=c(0.3,5),breaks=c(0.5,1,2,4))+theme_classic(base_size=13)+ annotate("text",label="P-overall < 0.001",x=85,y=4.5,colour="black",size=3.5)+ annotate("text",label="P-nonlinear < 0.251",x=85,y=3.5,colour="black",size=3.5)

#whr spline

coxmwhr<- fit.mult.impute(Surv(timetohf,hfany)~rcs(age,3)+rcs(irsadpercent,3)+rcs(alcoholqty,3)+ rcs(WHR_trim,3)+sex+smoker+hx_mi+hx_stroke+hx_ht+hx_chol+ hx_depression+hx_cabg+hx_stent+hx_arrhyth+hx_cad+hx_vd+hx_valve_dis+hx_kidney_dis+hx_osa+hx_asthma+hx_arthritis+hx_retinopathy +hx_migraine+hx_diab_any,fitter=cph,xtrans=impbkdata,data=bkdata)
coxmwhrlinear<- fit.mult.impute(Surv(timetohf,hfany)~rcs(age,3)+rcs(irsadpercent,3)+rcs(alcoholqty,3)+ WHR_trim+sex+smoker+hx_mi+hx_stroke+hx_ht+hx_chol+ hx_depression+hx_cabg+hx_stent+hx_arrhyth+hx_cad+hx_vd+hx_valve_dis+hx_kidney_dis+hx_osa+hx_asthma+hx_arthritis+hx_retinopathy +hx_migraine+hx_diab_any,fitter=cph,xtrans=impbkdata,data=bkdata)
coxmwhrno<- fit.mult.impute(Surv(timetohf,hfany)~rcs(age,3)+rcs(irsadpercent,3)+rcs(alcoholqty,3)+ sex+smoker+hx_mi+hx_stroke+hx_ht+hx_chol+ hx_depression+hx_cabg+hx_stent+hx_arrhyth+hx_cad+hx_vd+hx_valve_dis+hx_kidney_dis+hx_osa+hx_asthma+hx_arthritis+hx_retinopathy +hx_migraine+hx_diab_any,fitter=cph,xtrans=impbkdata,data=bkdata)
AIC(coxmwhr)
lrtest(coxmwhr,coxmwhrno)
lrtest(coxmwhr,coxmwhrlinear)
prcoxwhr<-Predict(coxmwhr,WHR_trim=seq(.77,1.1,by=.01),ref.zero=TRUE,fun=exp)
ggwhr<-ggplot(data=prcoxwhr,colfill='dodgerblue2',xlab="Waist-Hip Ratio",ylab="HR (95%CI)",adj.subtitle=FALSE)+geom_hline(aes(yintercept=1),linetype=3,col="black",size=.4)+geom_line(aes(x=WHR_trim,y=yhat),col='dodgerblue2',size=.75);
ggwhr$coordinates$limits$y <- NULL;
ggwhr+ scale_y_continuous(trans='log2',limits=c(0.3,5),breaks=c(0.5,1,2,4))+theme_classic(base_size=13)+ annotate("text",label="P-overall < 0.001",x=.85,y=4.5,colour="black",size=3.5)+ annotate("text",label="P-nonlinear = 0.001",x=.85,y=3.5,colour="black",size=3.5)


#sbp spline

coxmsbp_mean<- fit.mult.impute(Surv(timetohf,hfany)~rcs(sbp_mean,3)+rcs(age,3)+rcs(irsadpercent,3)+rcs(alcoholqty,3)+rcs(waist_trim,4)+rcs(BMI_trim,3)+sex+smoker+hx_mi+hx_stroke+hx_ht+hx_chol+ hx_depression+hx_cabg+hx_stent+hx_arrhyth+hx_cad+hx_vd+hx_valve_dis+hx_kidney_dis+hx_osa+hx_asthma+hx_arthritis+hx_retinopathy +hx_migraine+hx_diab_any+bpmed,fitter=cph,xtrans=impbkdata,data=bkdata)
coxmsbp_meanlinear<- fit.mult.impute(Surv(timetohf,hfany)~sbp_mean+rcs(age,3)+rcs(irsadpercent,3)+rcs(alcoholqty,3)+rcs(waist_trim,4)+rcs(BMI_trim,3)+sex+smoker+hx_mi+hx_stroke+hx_ht+hx_chol+ hx_depression+hx_cabg+hx_stent+hx_arrhyth+hx_cad+hx_vd+hx_valve_dis+hx_kidney_dis+hx_osa+hx_asthma+hx_arthritis+hx_retinopathy +hx_migraine+hx_diab_any+bpmed,fitter=cph,xtrans=impbkdata,data=bkdata)
coxmsbp_meanno<- fit.mult.impute(Surv(timetohf,hfany)~rcs(age,3)+rcs(irsadpercent,3)+rcs(alcoholqty,3)+rcs(waist_trim,4)+rcs(BMI_trim,3)+sex+smoker+hx_mi+hx_stroke+hx_ht+hx_chol+ hx_depression+hx_cabg+hx_stent+hx_arrhyth+hx_cad+hx_vd+hx_valve_dis+hx_kidney_dis+hx_osa+hx_asthma+hx_arthritis+hx_retinopathy +hx_migraine+hx_diab_any+bpmed,fitter=cph,xtrans=impbkdata,data=bkdata)
AIC(coxmsbp_mean)
lrtest(coxmsbp_mean,coxmsbp_meanno)
lrtest(coxmsbp_mean,coxmsbp_meanlinear)
prcoxsbp_mean<-Predict(coxmsbp_mean,sbp_mean=seq(90,200,by=1),ref.zero=TRUE,fun=exp)
ggsbp_mean<-ggplot(data=prcoxsbp_mean,colfill='dodgerblue2',xlab="Systolic Blood Pressure",ylab="HR (95%CI)",adj.subtitle=FALSE)+geom_hline(aes(yintercept=1),linetype=3,col="black",size=.4)+geom_line(aes(x=sbp_mean,y=yhat),col='dodgerblue2',size=.75);
ggsbp_mean$coordinates$limits$y <- NULL;
ggsbp_mean+ scale_y_continuous(trans='log2',limits=c(0.3,5),breaks=c(0.5,1,2,4))+theme_classic(base_size=13)+ annotate("text",label="P-overall = 0.013",x=120,y=4.5,colour="black",size=3.5)+ annotate("text",label="P-nonlinear = 0.014",x=120,y=3.5,colour="black",size=3.5)

#dbp spline

coxmdbp_mean<- fit.mult.impute(Surv(timetohf,hfany)~rcs(dbp_mean,3)+rcs(age,3)+rcs(irsadpercent,3)+rcs(alcoholqty,3)+rcs(waist_trim,4)+rcs(BMI_trim,3)+sex+smoker+hx_mi+hx_stroke+hx_ht+hx_chol+ hx_depression+hx_cabg+hx_stent+hx_arrhyth+hx_cad+hx_vd+hx_valve_dis+hx_kidney_dis+hx_osa+hx_asthma+hx_arthritis+hx_retinopathy +hx_migraine+hx_diab_any+bpmed,fitter=cph,xtrans=impbkdata,data=bkdata)
coxmdbp_meanlinear<- fit.mult.impute(Surv(timetohf,hfany)~dbp_mean+rcs(age,3)+rcs(irsadpercent,3)+rcs(alcoholqty,3)+rcs(waist_trim,4)+rcs(BMI_trim,3)+sex+smoker+hx_mi+hx_stroke+hx_ht+hx_chol+ hx_depression+hx_cabg+hx_stent+hx_arrhyth+hx_cad+hx_vd+hx_valve_dis+hx_kidney_dis+hx_osa+hx_asthma+hx_arthritis+hx_retinopathy +hx_migraine+hx_diab_any+bpmed,fitter=cph,xtrans=impbkdata,data=bkdata)
coxmdbp_meanno<- fit.mult.impute(Surv(timetohf,hfany)~ rcs(age,3)+rcs(irsadpercent,3)+rcs(alcoholqty,3)+rcs(waist_trim,4)+rcs(BMI_trim,3)+sex+smoker+hx_mi+hx_stroke+hx_ht+hx_chol+ hx_depression+hx_cabg+hx_stent+hx_arrhyth+hx_cad+hx_vd+hx_valve_dis+hx_kidney_dis+hx_osa+hx_asthma+hx_arthritis+hx_retinopathy +hx_migraine+hx_diab_any+bpmed,fitter=cph,xtrans=impbkdata,data=bkdata)
AIC(coxmdbp_mean)
lrtest(coxmdbp_mean,coxmdbp_meanno)
lrtest(coxmdbp_mean,coxmdbp_meanlinear)
prcoxdbp_mean<-Predict(coxmdbp_mean,dbp_mean=seq(50,110,by=1),ref.zero=TRUE,fun=exp)
ggdbp_mean<-ggplot(data=prcoxdbp_mean,colfill='dodgerblue2',xlab="Diastolic Blood Pressure",ylab="HR (95%CI)",adj.subtitle=FALSE)+geom_hline(aes(yintercept=1),linetype=3,col="black",size=.4)+geom_line(aes(x=dbp_mean,y=yhat),col='dodgerblue2',size=.75);
ggdbp_mean$coordinates$limits$y <- NULL;
ggdbp_mean+ scale_y_continuous(trans='log2',limits=c(0.3,5),breaks=c(0.5,1,2,4))+theme_classic(base_size=13)+ annotate("text",label="P-overall = 0.344",x=66,y=4.5,colour="black",size=3.5)+ annotate("text",label="P-nonlinear = 0.676",x=66,y=3.5,colour="black",size=3.5)

#glucose spline

coxmgluc<- fit.mult.impute(Surv(timetohf,hfany)~ rcs(gluc,4) +rcs(age,3)+rcs(irsadpercent,3)+rcs(alcoholqty,3)+rcs(waist_trim,4)+rcs(BMI_trim,3)+sex+smoker+hx_mi+hx_stroke+hx_ht+hx_chol+ hx_depression+hx_cabg+hx_stent+hx_arrhyth+hx_cad+hx_vd+hx_valve_dis+hx_kidney_dis+hx_osa+hx_asthma+hx_arthritis+hx_retinopathy +hx_migraine+hx_diab_any+dmmed,fitter=cph,xtrans=impbkdata,data=bkdata)
coxmgluclinear<- fit.mult.impute(Surv(timetohf,hfany)~ gluc+rcs(age,3)+rcs(irsadpercent,3)+rcs(alcoholqty,3)+rcs(waist_trim,4)+rcs(BMI_trim,3)+sex+smoker+hx_mi+hx_stroke+hx_ht+hx_chol+ hx_depression+hx_cabg+hx_stent+hx_arrhyth+hx_cad+hx_vd+hx_valve_dis+hx_kidney_dis+hx_osa+hx_asthma+hx_arthritis+hx_retinopathy +hx_migraine+hx_diab_any+dmmed,fitter=cph,xtrans=impbkdata,data=bkdata)
coxmglucno<- fit.mult.impute(Surv(timetohf,hfany)~ rcs(age,3)+rcs(irsadpercent,3)+rcs(alcoholqty,3)+rcs(waist_trim,4)+rcs(BMI_trim,3)+sex+smoker+hx_mi+hx_stroke+hx_ht+hx_chol+ hx_depression+hx_cabg+hx_stent+hx_arrhyth+hx_cad+hx_vd+hx_valve_dis+hx_kidney_dis+hx_osa+hx_asthma+hx_arthritis+hx_retinopathy +hx_migraine+hx_diab_any+dmmed,fitter=cph,xtrans=impbkdata,data=bkdata)
AIC(coxmgluc)
lrtest(coxmgluc,coxmglucno)
lrtest(coxmgluc,coxmgluclinear)
prcoxgluc<-Predict(coxmgluc,gluc=seq(3,20,by=.1),ref.zero=TRUE,fun=exp)
gggluc<-ggplot(data=prcoxgluc,colfill='dodgerblue2',xlab="Fasting Glucose",ylab="HR (95%CI)",adj.subtitle=FALSE)+geom_hline(aes(yintercept=1),linetype=3,col="black",size=.4)+geom_line(aes(x=gluc,y=yhat),col='dodgerblue2',size=.75);
gggluc$coordinates$limits$y <- NULL;
gggluc+ scale_y_continuous(trans='log2',limits=c(0.3,5),breaks=c(0.5,1,2,4))+theme_classic(base_size=13)+ annotate("text",label="P-overall = 0.070",x=7,y=4.5,colour="black",size=3.5)+ annotate("text",label="P-nonlinear = 0.167",x=7,y=3.5,colour="black",size=3.5)


#ldl spline

coxmldl<- fit.mult.impute(Surv(timetohf,hfany)~ rcs(ldl,4) +rcs(age,3)+rcs(irsadpercent,3)+rcs(alcoholqty,3)+rcs(waist_trim,4)+rcs(BMI_trim,3)+sex+smoker+hx_mi+hx_stroke+hx_ht+hx_chol+ hx_depression+hx_cabg+hx_stent+hx_arrhyth+hx_cad+hx_vd+hx_valve_dis+hx_kidney_dis+hx_osa+hx_asthma+hx_arthritis+hx_retinopathy +hx_migraine+hx_diab_any+cholmed,fitter=cph,xtrans=impbkdata,data=bkdata)
coxmldllinear<- fit.mult.impute(Surv(timetohf,hfany)~ ldl+rcs(age,3)+rcs(irsadpercent,3)+rcs(alcoholqty,3)+rcs(waist_trim,4)+rcs(BMI_trim,3)+sex+smoker+hx_mi+hx_stroke+hx_ht+hx_chol+ hx_depression+hx_cabg+hx_stent+hx_arrhyth+hx_cad+hx_vd+hx_valve_dis+hx_kidney_dis+hx_osa+hx_asthma+hx_arthritis+hx_retinopathy +hx_migraine+hx_diab_any+cholmed,fitter=cph,xtrans=impbkdata,data=bkdata)
coxmldlno<- fit.mult.impute(Surv(timetohf,hfany)~ rcs(age,3)+rcs(irsadpercent,3)+rcs(alcoholqty,3)+rcs(waist_trim,4)+rcs(BMI_trim,3)+sex+smoker+hx_mi+hx_stroke+hx_ht+hx_chol+ hx_depression+hx_cabg+hx_stent+hx_arrhyth+hx_cad+hx_vd+hx_valve_dis+hx_kidney_dis+hx_osa+hx_asthma+hx_arthritis+hx_retinopathy +hx_migraine+hx_diab_any+cholmed,fitter=cph,xtrans=impbkdata,data=bkdata)
AIC(coxmldl)
lrtest(coxmldl,coxmldlno)
lrtest(coxmldl,coxmldllinear)
prcoxldl<-Predict(coxmldl,ldl=seq(1,5,by=.1),ref.zero=TRUE,fun=exp)
ggldl<-ggplot(data=prcoxldl,colfill='dodgerblue2',xlab="LDL-C",ylab="HR (95%CI)",adj.subtitle=FALSE)+geom_hline(aes(yintercept=1),linetype=3,col="black",size=.4)+geom_line(aes(x=ldl,y=yhat),col='dodgerblue2',size=.75);
ggldl$coordinates$limits$y <- NULL;
ggldl+ scale_y_continuous(trans='log2',limits=c(0.3,5),breaks=c(0.5,1,2,4))+theme_classic(base_size=13)+ annotate("text",label="P-overall < 0.001",x=1.8,y=4.5,colour="black",size=3.5)+ annotate("text",label="P-nonlinear = 0.130",x=1.8,y=3.5,colour="black",size=3.5)

#hdl spline

coxmhdl<- fit.mult.impute(Surv(timetohf,hfany)~ rcs(hdl,3)+ rcs(age,3)+rcs(irsadpercent,3)+rcs(alcoholqty,3)+rcs(waist_trim,4)+rcs(BMI_trim,3)+sex+smoker+hx_mi+hx_stroke+hx_ht+hx_chol+ hx_depression+hx_cabg+hx_stent+hx_arrhyth+hx_cad+hx_vd+hx_valve_dis+hx_kidney_dis+hx_osa+hx_asthma+hx_arthritis+hx_retinopathy +hx_migraine+hx_diab_any+cholmed,fitter=cph,xtrans=impbkdata,data=bkdata)
coxmhdllinear<- fit.mult.impute(Surv(timetohf,hfany)~ hdl +rcs(age,3)+rcs(irsadpercent,3)+rcs(alcoholqty,3)+rcs(waist_trim,4)+rcs(BMI_trim,3)+sex+smoker+hx_mi+hx_stroke+hx_ht+hx_chol+ hx_depression+hx_cabg+hx_stent+hx_arrhyth+hx_cad+hx_vd+hx_valve_dis+hx_kidney_dis+hx_osa+hx_asthma+hx_arthritis+hx_retinopathy +hx_migraine+hx_diab_any+cholmed,fitter=cph,xtrans=impbkdata,data=bkdata)
coxmhdlno<- fit.mult.impute(Surv(timetohf,hfany)~ rcs(age,3)+rcs(irsadpercent,3)+rcs(alcoholqty,3)+rcs(waist_trim,4)+rcs(BMI_trim,3)+sex+smoker+hx_mi+hx_stroke+hx_ht+hx_chol+ hx_depression+hx_cabg+hx_stent+hx_arrhyth+hx_cad+hx_vd+hx_valve_dis+hx_kidney_dis+hx_osa+hx_asthma+hx_arthritis+hx_retinopathy +hx_migraine+hx_diab_any+cholmed,fitter=cph,xtrans=impbkdata,data=bkdata)
AIC(coxmhdl)
lrtest(coxmhdl,coxmhdlno)
lrtest(coxmhdl,coxmhdllinear)
prcoxhdl<-Predict(coxmhdl,hdl=seq(.1,2.5,by=.1),ref.zero=TRUE,fun=exp)
gghdl<-ggplot(data=prcoxhdl,colfill='dodgerblue2',xlab="HDL-C",ylab="HR (95%CI)",adj.subtitle=FALSE)+geom_hline(aes(yintercept=1),linetype=3,col="black",size=.4)+geom_line(aes(x=hdl,y=yhat),col='dodgerblue2',size=.75);
gghdl$coordinates$limits$y <- NULL;
gghdl+ scale_y_continuous(trans='log2',limits=c(0.3,5),breaks=c(0.5,1,2,4))+theme_classic(base_size=13)+ annotate("text",label="P-overall = 0.013",x=.7,y=4.5,colour="black",size=3.5)+ annotate("text",label="P-nonlinear = 0.006",x=.7,y=3.5,colour="black",size=3.5)

#trigs spline

coxmtrigs<- fit.mult.impute(Surv(timetohf,hfany)~ rcs(trigs,3) +rcs(age,3)+rcs(irsadpercent,3)+rcs(alcoholqty,3)+rcs(waist_trim,4)+rcs(BMI_trim,3)+sex+smoker+hx_mi+hx_stroke+hx_ht+hx_chol+ hx_depression+hx_cabg+hx_stent+hx_arrhyth+hx_cad+hx_vd+hx_valve_dis+hx_kidney_dis+hx_osa+hx_asthma+hx_arthritis+hx_retinopathy +hx_migraine+hx_diab_any+cholmed,fitter=cph,xtrans=impbkdata,data=bkdata)
coxmtrigslinear<- fit.mult.impute(Surv(timetohf,hfany)~ trigs+rcs(age,3)+rcs(irsadpercent,3)+rcs(alcoholqty,3)+rcs(waist_trim,4)+rcs(BMI_trim,3)+sex+smoker+hx_mi+hx_stroke+hx_ht+hx_chol+ hx_depression+hx_cabg+hx_stent+hx_arrhyth+hx_cad+hx_vd+hx_valve_dis+hx_kidney_dis+hx_osa+hx_asthma+hx_arthritis+hx_retinopathy +hx_migraine+hx_diab_any+cholmed,fitter=cph,xtrans=impbkdata,data=bkdata)
coxmtrigsno<- fit.mult.impute(Surv(timetohf,hfany)~ rcs(age,3)+rcs(irsadpercent,3)+rcs(alcoholqty,3)+rcs(waist_trim,4)+rcs(BMI_trim,3)+sex+smoker+hx_mi+hx_stroke+hx_ht+hx_chol+ hx_depression+hx_cabg+hx_stent+hx_arrhyth+hx_cad+hx_vd+hx_valve_dis+hx_kidney_dis+hx_osa+hx_asthma+hx_arthritis+hx_retinopathy +hx_migraine+hx_diab_any+cholmed,fitter=cph,xtrans=impbkdata,data=bkdata)
AIC(coxmtrigs)
lrtest(coxmtrigs,coxmtrigsno)
lrtest(coxmtrigs,coxmtrigslinear)
prcoxtrigs<-Predict(coxmtrigs,trigs=seq(0,10,by=.1),ref.zero=TRUE,fun=exp)
ggtrigs<-ggplot(data=prcoxtrigs,colfill='dodgerblue2',xlab="Triglylcerides",ylab="HR (95%CI)",adj.subtitle=FALSE)+geom_hline(aes(yintercept=1),linetype=3,col="black",size=.4)+geom_line(aes(x=trigs,y=yhat),col='dodgerblue2',size=.75);
ggtrigs$coordinates$limits$y <- NULL;
ggtrigs+ scale_y_continuous(trans='log2',limits=c(0.3,5),breaks=c(0.5,1,2,4))+theme_classic(base_size=13)+ annotate("text",label="P-overall = 0.773",x=2,y=4.5,colour="black",size=3.5)+ annotate("text",label="P-nonlinear = 0.686",x=2,y=3.5,colour="black",size=3.5)


#Cox interactions between ASCVD and no-ASCVD, analysis for Figure 5

#age interaction

coxmageint<- fit.mult.impute(Surv(timetohf,hfany)~rcs(sbp_mean,3)+rcs(age,3)*ascvd+rcs(irsadpercent,3)+rcs(alcoholqty,3)+rcs(waist_trim,4)+rcs(BMI_trim,3)+sex+smoker+hx_ht+hx_chol+ hx_depression+hx_arrhyth+hx_valve_dis+hx_kidney_dis+hx_osa+hx_asthma+hx_arthritis+hx_retinopathy +hx_migraine+hx_diab_any,fitter=cph,xtrans=impbkdata,data=bkdata)
coxmagenoint<- fit.mult.impute(Surv(timetohf,hfany)~rcs(sbp_mean,3)+rcs(age,3)+ascvd+rcs(irsadpercent,3)+rcs(alcoholqty,3)+rcs(waist_trim,4)+rcs(BMI_trim,3)+sex+smoker+hx_ht+hx_chol+ hx_depression+hx_arrhyth+hx_valve_dis+hx_kidney_dis+hx_osa+hx_asthma+hx_arthritis+hx_retinopathy +hx_migraine+hx_diab_any,fitter=cph,xtrans=impbkdata,data=bkdata)
lrtest(coxmageint,coxmagenoint)
cspline1age<-Predict(coxmageint,age=seq(35,85,by=.1),ref.zero=TRUE,fun=exp,ascvd=0);
cspline2age<-Predict(coxmageint,age=seq(35,85,by=.1),ref.zero=TRUE,fun=exp,ascvd=1);
cspline1age$coordinates$limits$y<-NULL;
cspline2age$coordinates$limits$y<-NULL;
ggage<-ggplot()+geom_line(data=cspline1age,aes(x=age,y=yhat),size=1 ,col='blue3')+geom_line(data=cspline2age,aes(x=age,y=yhat),col='firebrick3',size=1)+geom_hline(aes(yintercept=1),linetype=3,col="black",size=.4)
ggage$coordinates$limits$y <- NULL;
ggage +scale_y_continuous(trans='log2',limits=c(0.3,50),breaks=c(0.5,1,2,4,8,16,32))+theme_classic(base_size=13) + annotate("text",label="P-interaction = 0.366",x=50,y=32,colour="black",size=3.5)+ labs(y="HR",x="Age")

#ses interaction

coxmirsadpercentint<- fit.mult.impute(Surv(timetohf,hfany)~rcs(gluc,3)+rcs(ldl,3)+rcs(sbp_mean,3)+rcs(age,3)+ascvd*rcs(irsadpercent,3)+rcs(alcoholqty,3)+rcs(waist_trim,4)+rcs(BMI_trim,3)+sex+smoker+hx_ht+hx_chol+ hx_depression+hx_arrhyth+hx_valve_dis+hx_kidney_dis+hx_osa+hx_asthma+hx_arthritis+hx_retinopathy +hx_migraine+hx_diab_any,fitter=cph,xtrans=impbkdata,data=bkdata)
coxmirsadpercentnoint<- fit.mult.impute(Surv(timetohf,hfany)~rcs(gluc,3)+rcs(ldl,3)+rcs(sbp_mean,3)+rcs(age,3)+ascvd+rcs(irsadpercent,3)+rcs(alcoholqty,3)+rcs(waist_trim,4)+rcs(BMI_trim,3)+sex+smoker+hx_ht+hx_chol+ hx_depression+hx_arrhyth+hx_valve_dis+hx_kidney_dis+hx_osa+hx_asthma+hx_arthritis+hx_retinopathy +hx_migraine+hx_diab_any,fitter=cph,xtrans=impbkdata,data=bkdata)
lrtest(coxmirsadpercentint,coxmirsadpercentnoint)
cspline1irsadpercent<-Predict(coxmirsadpercentint,irsadpercent=seq(0,100,by=.1),ref.zero=TRUE,fun=exp,ascvd=0);
cspline2irsadpercent<-Predict(coxmirsadpercentint,irsadpercent=seq(0,100,by=.1),ref.zero=TRUE,fun=exp,ascvd=1);
cspline1irsadpercent$coordinates$limits$y<-NULL;
cspline2irsadpercent$coordinates$limits$y<-NULL;
ggirsadpercent<-ggplot()+geom_line(data=cspline1irsadpercent,aes(x=irsadpercent,y=yhat),size=1 ,col='blue3')+geom_line(data=cspline2irsadpercent,aes(x=irsadpercent,y=yhat),col='firebrick3',size=1)+geom_hline(aes(yintercept=1),linetype=3,col="black",size=.4)
ggirsadpercent$coordinates$limits$y <- NULL;
ggirsadpercent +scale_y_continuous(trans='log2',limits=c(0.3,20),breaks=c(0.5,1,2,4,8,16))+theme_classic(base_size=13) + annotate("text",label="P-interaction = 0.385",x=25,y=16,colour="black",size=3.5)+ labs(y="HR",x="Socioeconomic Status")

#alcohol interaction

coxmalcoholqtyint<- fit.mult.impute(Surv(timetohf,hfany)~rcs(gluc,3)+rcs(ldl,3)+rcs(sbp_mean,3)+rcs(age,3)+rcs(irsadpercent,3)+rcs(alcoholqty,3)*ascvd+rcs(waist_trim,4)+rcs(BMI_trim,3)+sex+smoker+hx_ht+hx_chol+ hx_depression+hx_arrhyth+hx_valve_dis+hx_kidney_dis+hx_osa+hx_asthma+hx_arthritis+hx_retinopathy +hx_migraine+hx_diab_any,fitter=cph,xtrans=impbkdata,data=bkdata)
coxmalcoholqtynoint<- fit.mult.impute(Surv(timetohf,hfany)~rcs(gluc,3)+rcs(ldl,3)+rcs(sbp_mean,3)+rcs(age,3)+ascvd+rcs(irsadpercent,3)+rcs(alcoholqty,3)+rcs(waist_trim,4)+rcs(BMI_trim,3)+sex+smoker+hx_ht+hx_chol+ hx_depression+hx_arrhyth+hx_valve_dis+hx_kidney_dis+hx_osa+hx_asthma+hx_arthritis+hx_retinopathy +hx_migraine+hx_diab_any,fitter=cph,xtrans=impbkdata,data=bkdata)
lrtest(coxmalcoholqtyint,coxmalcoholqtynoint)
cspline1alcoholqty<-Predict(coxmalcoholqtyint,alcoholqty=seq(0,42,by=.1),ref.zero=TRUE,fun=exp,ascvd=0);
cspline2alcoholqty<-Predict(coxmalcoholqtyint,alcoholqty=seq(0,42,by=.1),ref.zero=TRUE,fun=exp,ascvd=1);
cspline1alcoholqty$coordinates$limits$y<-NULL;
cspline2alcoholqty$coordinates$limits$y<-NULL;
ggalcoholqty<-ggplot()+geom_line(data=cspline1alcoholqty,aes(x=alcoholqty,y=yhat),size=1 ,col='blue3')+geom_line(data=cspline2alcoholqty,aes(x=alcoholqty,y=yhat),col='firebrick3',size=1)+geom_hline(aes(yintercept=1),linetype=3,col="black",size=.4)
ggalcoholqty$coordinates$limits$y <- NULL;
ggalcoholqty +scale_y_continuous(trans='log2',limits=c(0.3,20),breaks=c(0.5,1,2,4,8,16))+theme_classic(base_size=13) + annotate("text",label="P-interaction = 0.496",x=10,y=16,colour="black",size=3.5)+ labs(y="HR",x="Alcohol (SD per week)")

#bmi interaction

coxmBMI_trimint<- fit.mult.impute(Surv(timetohf,hfany)~rcs(gluc,3)+rcs(ldl,3)+rcs(sbp_mean,3)+rcs(age,3)+rcs(irsadpercent,3)+rcs(alcoholqty,3)+rcs(waist_trim,4)+rcs(BMI_trim,3)*ascvd+sex+smoker+hx_ht+hx_chol+ hx_depression+hx_arrhyth+hx_valve_dis+hx_kidney_dis+hx_osa+hx_asthma+hx_arthritis+hx_retinopathy +hx_migraine+hx_diab_any,fitter=cph,xtrans=impbkdata,data=bkdata)
coxmBMI_trimnoint<- fit.mult.impute(Surv(timetohf,hfany)~rcs(gluc,3)+rcs(ldl,3)+rcs(sbp_mean,3)+rcs(age,3)+ascvd+rcs(irsadpercent,3)+rcs(alcoholqty,3)+rcs(waist_trim,4)+rcs(BMI_trim,3)+sex+smoker+hx_ht+hx_chol+ hx_depression+hx_arrhyth+hx_valve_dis+hx_kidney_dis+hx_osa+hx_asthma+hx_arthritis+hx_retinopathy +hx_migraine+hx_diab_any,fitter=cph,xtrans=impbkdata,data=bkdata)
lrtest(coxmBMI_trimint,coxmBMI_trimnoint)
cspline1BMI_trim<-Predict(coxmBMI_trimint,BMI_trim=seq(20,40,by=.1),ref.zero=TRUE,fun=exp,ascvd=0);
cspline2BMI_trim<-Predict(coxmBMI_trimint,BMI_trim=seq(20,40,by=.1),ref.zero=TRUE,fun=exp,ascvd=1);
cspline1BMI_trim$coordinates$limits$y<-NULL;
cspline2BMI_trim$coordinates$limits$y<-NULL;
ggBMI_trim<-ggplot()+geom_line(data=cspline1BMI_trim,aes(x=BMI_trim,y=yhat),size=1 ,col='blue3')+geom_line(data=cspline2BMI_trim,aes(x=BMI_trim,y=yhat),col='firebrick3',size=1)+geom_hline(aes(yintercept=1),linetype=3,col="black",size=.4)
ggBMI_trim$coordinates$limits$y <- NULL;
ggBMI_trim +scale_y_continuous(trans='log2',limits=c(0.3,20),breaks=c(0.5,1,2,4,8,16))+theme_classic(base_size=13) + annotate("text",label="P-interaction = 0.465",x=25,y=16,colour="black",size=3.5)+ labs(y="HR",x="Body Mass Index")

#waist interaction

coxmwaist_trimint<- fit.mult.impute(Surv(timetohf,hfany)~rcs(gluc,3)+rcs(ldl,3)+rcs(sbp_mean,3)+rcs(age,3)+rcs(irsadpercent,3)+rcs(alcoholqty,3)+rcs(waist_trim,4)*ascvd+rcs(BMI_trim,3)+sex+smoker+hx_ht+hx_chol+ hx_depression+hx_arrhyth+hx_valve_dis+hx_kidney_dis+hx_osa+hx_asthma+hx_arthritis+hx_retinopathy +hx_migraine+hx_diab_any,fitter=cph,xtrans=impbkdata,data=bkdata)
coxmwaist_trimnoint<- fit.mult.impute(Surv(timetohf,hfany)~rcs(gluc,3)+rcs(ldl,3)+rcs(sbp_mean,3)+rcs(age,3)+ascvd+rcs(irsadpercent,3)+rcs(alcoholqty,3)+rcs(waist_trim,4)+rcs(BMI_trim,3)+sex+smoker+hx_ht+hx_chol+ hx_depression+hx_arrhyth+hx_valve_dis+hx_kidney_dis+hx_osa+hx_asthma+hx_arthritis+hx_retinopathy +hx_migraine+hx_diab_any,fitter=cph,xtrans=impbkdata,data=bkdata)
lrtest(coxmwaist_trimint,coxmwaist_trimnoint)
cspline1waist_trim<-Predict(coxmwaist_trimint,waist_trim=seq(75,125,by=.1),ref.zero=TRUE,fun=exp,ascvd=0);
cspline2waist_trim<-Predict(coxmwaist_trimint,waist_trim=seq(75,125,by=.1),ref.zero=TRUE,fun=exp,ascvd=1);
cspline1waist_trim$coordinates$limits$y<-NULL;
cspline2waist_trim$coordinates$limits$y<-NULL;
ggwaist_trim<-ggplot()+geom_line(data=cspline1waist_trim,aes(x=waist_trim,y=yhat),size=1 ,col='blue3')+geom_line(data=cspline2waist_trim,aes(x=waist_trim,y=yhat),col='firebrick3',size=1)+geom_hline(aes(yintercept=1),linetype=3,col="black",size=.4)
ggwaist_trim$coordinates$limits$y <- NULL;
ggwaist_trim +scale_y_continuous(trans='log2',limits=c(0.3,20),breaks=c(0.5,1,2,4,8,16))+theme_classic(base_size=13) + annotate("text",label="P-interaction = 0.115",x=90,y=16,colour="black",size=3.5)+ labs(y="HR",x="Waist Circumference")

#WHR interaction

coxmWHR_trimint<- fit.mult.impute(Surv(timetohf,hfany)~rcs(gluc,3)+rcs(ldl,3)+rcs(sbp_mean,3)+rcs(age,3)+rcs(irsadpercent,3)+rcs(alcoholqty,3)+rcs(WHR_trim,3)*ascvd+rcs(BMI_trim,3)+sex+smoker+hx_ht+hx_chol+ hx_depression+hx_arrhyth+hx_valve_dis+hx_kidney_dis+hx_osa+hx_asthma+hx_arthritis+hx_retinopathy +hx_migraine+hx_diab_any,fitter=cph,xtrans=impbkdata,data=bkdata)
coxmWHR_trimnoint<- fit.mult.impute(Surv(timetohf,hfany)~rcs(gluc,3)+rcs(ldl,3)+rcs(sbp_mean,3)+rcs(age,3)+ascvd+rcs(irsadpercent,3)+rcs(alcoholqty,3)+rcs(WHR_trim,3)+rcs(BMI_trim,3)+sex+smoker+hx_ht+hx_chol+ hx_depression+hx_arrhyth+hx_valve_dis+hx_kidney_dis+hx_osa+hx_asthma+hx_arthritis+hx_retinopathy +hx_migraine+hx_diab_any,fitter=cph,xtrans=impbkdata,data=bkdata)
lrtest(coxmWHR_trimint,coxmWHR_trimnoint)
cspline1WHR_trim<-Predict(coxmWHR_trimint,WHR_trim=seq(0.75,1.1,by=.01),ref.zero=TRUE,fun=exp,ascvd=0);
cspline2WHR_trim<-Predict(coxmWHR_trimint,WHR_trim=seq(0.75,1.1,by=.01),ref.zero=TRUE,fun=exp,ascvd=1);
cspline1WHR_trim$coordinates$limits$y<-NULL;
cspline2WHR_trim$coordinates$limits$y<-NULL;
ggWHR_trim<-ggplot()+geom_line(data=cspline1WHR_trim,aes(x=WHR_trim,y=yhat),size=1 ,col='blue3')+geom_line(data=cspline2WHR_trim,aes(x=WHR_trim,y=yhat),col='firebrick3',size=1)+geom_hline(aes(yintercept=1),linetype=3,col="black",size=.4)
ggWHR_trim$coordinates$limits$y <- NULL;
ggWHR_trim +scale_y_continuous(trans='log2',limits=c(0.3,20),breaks=c(0.5,1,2,4,8,16))+theme_classic(base_size=13) + annotate("text",label="P-interaction = 0.054",x=.85,y=16,colour="black",size=3.5)+ labs(y="HR",x="Waist-Hip Ratio")

#sbp interaction

coxmsbp_meanint<- fit.mult.impute(Surv(timetohf,hfany)~rcs(gluc,3)+rcs(ldl,3)+rcs(sbp_mean,3)*ascvd+rcs(age,3)+rcs(irsadpercent,3)+rcs(alcoholqty,3)+rcs(waist_trim,4)+rcs(BMI_trim,3)+sex+smoker+hx_ht+hx_chol+ hx_depression+hx_arrhyth+hx_valve_dis+hx_kidney_dis+hx_osa+hx_asthma+hx_arthritis+hx_retinopathy +hx_migraine+hx_diab_any+bpmed,fitter=cph,xtrans=impbkdata,data=bkdata)
coxmsbp_meannoint<- fit.mult.impute(Surv(timetohf,hfany)~rcs(gluc,3)+rcs(ldl,3)+rcs(sbp_mean,3)+ascvd+rcs(age,3)+rcs(irsadpercent,3)+rcs(alcoholqty,3)+rcs(waist_trim,4)+rcs(BMI_trim,3)+sex+smoker+hx_ht+hx_chol+ hx_depression+hx_arrhyth+hx_valve_dis+hx_kidney_dis+hx_osa+hx_asthma+hx_arthritis+hx_retinopathy +hx_migraine+hx_diab_any+bpmed,fitter=cph,xtrans=impbkdata,data=bkdata)
lrtest(coxmsbp_meanint,coxmsbp_meannoint)
cspline1sbp_mean<-Predict(coxmsbp_meanint,sbp_mean=seq(85,190,by=.1),ref.zero=TRUE,fun=exp,ascvd=0);
cspline2sbp_mean<-Predict(coxmsbp_meanint,sbp_mean=seq(85,190,by=.1),ref.zero=TRUE,fun=exp,ascvd=1);
cspline1sbp_mean$coordinates$limits$y<-NULL;
cspline2sbp_mean$coordinates$limits$y<-NULL;
ggsbp_mean<-ggplot()+geom_line(data=cspline1sbp_mean,aes(x=sbp_mean,y=yhat),size=1 ,col='blue3')+geom_line(data=cspline2sbp_mean,aes(x=sbp_mean,y=yhat),col='firebrick3',size=1)+geom_hline(aes(yintercept=1),linetype=3,col="black",size=.4)
ggsbp_mean$coordinates$limits$y <- NULL;
ggsbp_mean +scale_y_continuous(trans='log2',limits=c(0.3,20),breaks=c(0.5,1,2,4,8,16))+theme_classic(base_size=13) + annotate("text",label="P-interaction = 0.644",x=115,y=16,colour="black",size=3.5)+ labs(y="HR",x="Systolic blood pressure")

#dbp interaction

coxmdbp_meanint<- fit.mult.impute(Surv(timetohf,hfany)~rcs(gluc,3)+rcs(ldl,3)+rcs(dbp_mean,3)*ascvd+rcs(age,3)+rcs(irsadpercent,3)+rcs(alcoholqty,3)+rcs(waist_trim,4)+rcs(BMI_trim,3)+sex+smoker+hx_ht+hx_chol+ hx_depression+hx_arrhyth+hx_valve_dis+hx_kidney_dis+hx_osa+hx_asthma+hx_arthritis+hx_retinopathy +hx_migraine+hx_diab_any+bpmed,fitter=cph,xtrans=impbkdata,data=bkdata)
coxmdbp_meannoint<- fit.mult.impute(Surv(timetohf,hfany)~rcs(gluc,3)+rcs(ldl,3)+rcs(dbp_mean,3)+ascvd+rcs(age,3)+rcs(irsadpercent,3)+rcs(alcoholqty,3)+rcs(waist_trim,4)+rcs(BMI_trim,3)+sex+smoker+hx_ht+hx_chol+ hx_depression+hx_arrhyth+hx_valve_dis+hx_kidney_dis+hx_osa+hx_asthma+hx_arthritis+hx_retinopathy +hx_migraine+hx_diab_any+bpmed,fitter=cph,xtrans=impbkdata,data=bkdata)
lrtest(coxmdbp_meanint,coxmdbp_meannoint)
cspline1dbp_mean<-Predict(coxmdbp_meanint,dbp_mean=seq(55,105,by=.1),ref.zero=TRUE,fun=exp,ascvd=0);
cspline2dbp_mean<-Predict(coxmdbp_meanint,dbp_mean=seq(55,105,by=.1),ref.zero=TRUE,fun=exp,ascvd=1);
cspline1dbp_mean$coordinates$limits$y<-NULL;
cspline2dbp_mean$coordinates$limits$y<-NULL;
ggdbp_mean<-ggplot()+geom_line(data=cspline1dbp_mean,aes(x=dbp_mean,y=yhat),size=1 ,col='blue3')+geom_line(data=cspline2dbp_mean,aes(x=dbp_mean,y=yhat),col='firebrick3',size=1)+geom_hline(aes(yintercept=1),linetype=3,col="black",size=.4)
ggdbp_mean$coordinates$limits$y <- NULL;
ggdbp_mean +scale_y_continuous(trans='log2',limits=c(0.3,20),breaks=c(0.5,1,2,4,8,16))+theme_classic(base_size=13) + annotate("text",label="P-interaction = 0.693",x=70,y=16,colour="black",size=3.5)+ labs(y="HR",x="Diastolic blood pressure")

#Glucose interaction

coxmglucint<- fit.mult.impute(Surv(timetohf,hfany)~rcs(gluc,3)*ascvd+rcs(ldl,3)+rcs(sbp_mean,3)+rcs(age,3)+rcs(irsadpercent,3)+rcs(alcoholqty,3)+rcs(waist_trim,4)+rcs(BMI_trim,3)+sex+smoker+hx_ht+hx_chol+ hx_depression+hx_arrhyth+hx_valve_dis+hx_kidney_dis+hx_osa+hx_asthma+hx_arthritis+hx_retinopathy +hx_migraine+hx_diab_any+dmmed,fitter=cph,xtrans=impbkdata,data=bkdata)
coxmglucnoint<- fit.mult.impute(Surv(timetohf,hfany)~rcs(gluc,3)+rcs(ldl,3)+rcs(sbp_mean,3)+ascvd+rcs(age,3)+rcs(irsadpercent,3)+rcs(alcoholqty,3)+rcs(waist_trim,4)+rcs(BMI_trim,3)+sex+smoker+hx_ht+hx_chol+ hx_depression+hx_arrhyth+hx_valve_dis+hx_kidney_dis+hx_osa+hx_asthma+hx_arthritis+hx_retinopathy +hx_migraine+hx_diab_any+dmmed,fitter=cph,xtrans=impbkdata,data=bkdata)
lrtest(coxmglucint,coxmglucnoint)
cspline1gluc<-Predict(coxmglucint,gluc=seq(3,20,by=.1),ref.zero=TRUE,fun=exp,ascvd=0);
cspline2gluc<-Predict(coxmglucint,gluc=seq(3,20,by=.1),ref.zero=TRUE,fun=exp,ascvd=1);
cspline1gluc$coordinates$limits$y<-NULL;
cspline2gluc$coordinates$limits$y<-NULL;
gggluc<-ggplot()+geom_line(data=cspline1gluc,aes(x=gluc,y=yhat),size=1 ,col='blue3')+geom_line(data=cspline2gluc,aes(x=gluc,y=yhat),col='firebrick3',size=1)+geom_hline(aes(yintercept=1),linetype=3,col="black",size=.4)
gggluc$coordinates$limits$y <- NULL;
gggluc +scale_y_continuous(trans='log2',limits=c(0.3,20),breaks=c(0.5,1,2,4,8,16))+theme_classic(base_size=13) + annotate("text",label="P-interaction = 0.260",x=8,y=16,colour="black",size=3.5)+ labs(y="HR",x="Glucose")

#ldl interaction

coxmldlint<- fit.mult.impute(Surv(timetohf,hfany)~rcs(gluc,3)+rcs(ldl,3)*ascvd+rcs(sbp_mean,3)+rcs(age,3)+rcs(irsadpercent,3)+rcs(alcoholqty,3)+rcs(waist_trim,4)+rcs(BMI_trim,3)+sex+smoker+hx_ht+hx_chol+ hx_depression+hx_arrhyth+hx_valve_dis+hx_kidney_dis+hx_osa+hx_asthma+hx_arthritis+hx_retinopathy +hx_migraine+hx_diab_any+dmmed,fitter=cph,xtrans=impbkdata,data=bkdata)
coxmldlnoint<- fit.mult.impute(Surv(timetohf,hfany)~rcs(gluc,3)+rcs(ldl,3)+rcs(sbp_mean,3)+ascvd+rcs(age,3)+rcs(irsadpercent,3)+rcs(alcoholqty,3)+rcs(waist_trim,4)+rcs(BMI_trim,3)+sex+smoker+hx_ht+hx_chol+ hx_depression+hx_arrhyth+hx_valve_dis+hx_kidney_dis+hx_osa+hx_asthma+hx_arthritis+hx_retinopathy +hx_migraine+hx_diab_any+dmmed,fitter=cph,xtrans=impbkdata,data=bkdata)
lrtest(coxmldlint,coxmldlnoint)
cspline1ldl<-Predict(coxmldlint,ldl=seq(1,5,by=.1),ref.zero=TRUE,fun=exp,ascvd=0);
cspline2ldl<-Predict(coxmldlint,ldl=seq(1,5,by=.1),ref.zero=TRUE,fun=exp,ascvd=1);
cspline1ldl$coordinates$limits$y<-NULL;
cspline2ldl$coordinates$limits$y<-NULL;
ggldl<-ggplot()+geom_line(data=cspline1ldl,aes(x=ldl,y=yhat),size=1 ,col='blue3')+geom_line(data=cspline2ldl,aes(x=ldl,y=yhat),col='firebrick3',size=1)+geom_hline(aes(yintercept=1),linetype=3,col="black",size=.4)
ggldl$coordinates$limits$y <- NULL;
ggldl +scale_y_continuous(trans='log2',limits=c(0.3,20),breaks=c(0.5,1,2,4,8,16))+theme_classic(base_size=13) + annotate("text",label="P-interaction = 0.177",x=2.2,y=16,colour="black",size=3.5)+ labs(y="HR",x="LDL-C")

#hdl interaction

coxmhdlint<- fit.mult.impute(Surv(timetohf,hfany)~rcs(gluc,3)+rcs(hdl,3)*ascvd+rcs(sbp_mean,3)+rcs(age,3)+rcs(irsadpercent,3)+rcs(alcoholqty,3)+rcs(waist_trim,4)+rcs(BMI_trim,3)+sex+smoker+hx_ht+hx_chol+ hx_depression+hx_arrhyth+hx_valve_dis+hx_kidney_dis+hx_osa+hx_asthma+hx_arthritis+hx_retinopathy +hx_migraine+hx_diab_any+dmmed,fitter=cph,xtrans=impbkdata,data=bkdata)
coxmhdlnoint<- fit.mult.impute(Surv(timetohf,hfany)~rcs(gluc,3)+rcs(hdl,3)+rcs(sbp_mean,3)+ascvd+rcs(age,3)+rcs(irsadpercent,3)+rcs(alcoholqty,3)+rcs(waist_trim,4)+rcs(BMI_trim,3)+sex+smoker+hx_ht+hx_chol+ hx_depression+hx_arrhyth+hx_valve_dis+hx_kidney_dis+hx_osa+hx_asthma+hx_arthritis+hx_retinopathy +hx_migraine+hx_diab_any+dmmed,fitter=cph,xtrans=impbkdata,data=bkdata)
lrtest(coxmhdlint,coxmhdlnoint)
cspline1hdl<-Predict(coxmhdlint,hdl=seq(1,5,by=.1),ref.zero=TRUE,fun=exp,ascvd=0);
cspline2hdl<-Predict(coxmhdlint,hdl=seq(1,5,by=.1),ref.zero=TRUE,fun=exp,ascvd=1);
cspline1hdl$coordinates$limits$y<-NULL;
cspline2hdl$coordinates$limits$y<-NULL;
gghdl<-ggplot()+geom_line(data=cspline1hdl,aes(x=hdl,y=yhat),size=1 ,col='blue3')+geom_line(data=cspline2hdl,aes(x=hdl,y=yhat),col='firebrick3',size=1)+geom_hline(aes(yintercept=1),linetype=3,col="black",size=.4)
gghdl$coordinates$limits$y <- NULL;
gghdl +scale_y_continuous(trans='log2',limits=c(0.3,20),breaks=c(0.5,1,2,4,8,16))+theme_classic(base_size=13) + annotate("text",label="P-interaction = 0.676",x=2.2,y=16,colour="black",size=3.5)+ labs(y="HR",x="HDL-C")

#trigs interaction

coxmtrigsint<- fit.mult.impute(Surv(timetohf,hfany)~rcs(gluc,3)+rcs(trigs,3)*ascvd+rcs(sbp_mean,3)+rcs(age,3)+rcs(irsadpercent,3)+rcs(alcoholqty,3)+rcs(waist_trim,4)+rcs(BMI_trim,3)+sex+smoker+hx_ht+hx_chol+ hx_depression+hx_arrhyth+hx_valve_dis+hx_kidney_dis+hx_osa+hx_asthma+hx_arthritis+hx_retinopathy +hx_migraine+hx_diab_any+dmmed,fitter=cph,xtrans=impbkdata,data=bkdata)
coxmtrigsnoint<- fit.mult.impute(Surv(timetohf,hfany)~rcs(gluc,3)+rcs(trigs,3)+rcs(sbp_mean,3)+ascvd+rcs(age,3)+rcs(irsadpercent,3)+rcs(alcoholqty,3)+rcs(waist_trim,4)+rcs(BMI_trim,3)+sex+smoker+hx_ht+hx_chol+ hx_depression+hx_arrhyth+hx_valve_dis+hx_kidney_dis+hx_osa+hx_asthma+hx_arthritis+hx_retinopathy +hx_migraine+hx_diab_any+dmmed,fitter=cph,xtrans=impbkdata,data=bkdata)
lrtest(coxmtrigsint,coxmtrigsnoint)
cspline1trigs<-Predict(coxmtrigsint,trigs=seq(0,10,by=.1),ref.zero=TRUE,fun=exp,ascvd=0);
cspline2trigs<-Predict(coxmtrigsint,trigs=seq(0,10,by=.1),ref.zero=TRUE,fun=exp,ascvd=1);
cspline1trigs$coordinates$limits$y<-NULL;
cspline2trigs$coordinates$limits$y<-NULL;
ggtrigs<-ggplot()+geom_line(data=cspline1trigs,aes(x=trigs,y=yhat),size=1 ,col='blue3')+geom_line(data=cspline2trigs,aes(x=trigs,y=yhat),col='firebrick3',size=1)+geom_hline(aes(yintercept=1),linetype=3,col="black",size=.4)
ggtrigs$coordinates$limits$y <- NULL;
ggtrigs +scale_y_continuous(trans='log2',limits=c(0.3,20),breaks=c(0.5,1,2,4,8,16))+theme_classic(base_size=13) + annotate("text",label="P-interaction = 0.235",x=3,y=16,colour="black",size=3.5)+ labs(y="HR",x="Triglycerides")
